# Semi-field evaluation of the space spray efficacy of Fludora Co-Max EW against wild insecticide-resistant *Aedes aegypti* and *Culex quinquefasciatus* mosquito populations from Abidjan, Côte d’Ivoire

**DOI:** 10.1186/s13071-022-05572-5

**Published:** 2023-02-02

**Authors:** Julien Z. B. Zahouli, Jean-Denis Dibo, Fofana Diakaridia, Laurence V. A. Yao, Sarah D. Souza, Sebastian Horstmann, Benjamin G. Koudou

**Affiliations:** 1 Centre Suisse de Recherches Scientifiques en Côte d’Ivoire, Abidjan, Côte d’Ivoire; 2 Centre d’Entomologie Médicale et Vétérinaire, Université Alassane Ouattara, Bouaké, Côte d’Ivoire; 3 Unité de Formation et de Recherche Sciences de la Nature, Université Nangui-Abrogoua, Abidjan, Côte d’Ivoire; 4 Institut National d’Hygiène Publique, Ministère de la Santé et de l’Hygiène Publique, Abidjan, Côte d’Ivoire; 5 Envu, 2022 Environmental Science FR S.A.S., France, Lyon, France; 6 Envu, 2022 ES Deutschland GmbH, Germany, Monheim, Germany

**Keywords:** *Aedes aegypti*, *Culex quinquefasciatus*, Insecticide resistance, Fludora Co-Max EW, K-Othrine EC, Space spray, Arboviruses, Vector control, Côte d’Ivoire

## Abstract

**Background:**

Space spraying of insecticides is still an important means of controlling *Aedes* and *Culex* mosquitoes and arboviral diseases. This study evaluated the space spray efficacy of Fludora Co-Max EW, (water-based insecticide space spray combining flupyradifurone and transfluthrin with film forming aqueous spray technology (FFAST)), against wild insecticide-resistant *Aedes aegypti* and *Culex quinquefasciatus* mosquitoes from Abidjan, Côte d’Ivoire, compared with K-Othrine EC (deltamethrin-only product), in small-scale field trials.

**Methods:**

Wild *Ae. aegypti* and *Cx. quinquefasciatus* mosquito larvae were collected in Abidjan, Côte d’Ivoire from August to December 2020. Mosquito larvae were reared in the laboratory until the adult stage. Fludora Co-Max EW and K-Othrine EC were tested against emerged adult females (F_0_ generation) using ultra-low volume cold fogging (ULV) and thermal fogging (TF) delivery technology, both outdoors and indoors in Agboville, Côte d’Ivoire. Specifically, cages containing 20 mosquitoes each were placed at distances of 10, 25, 50, 75 and 100 m from the spraying line for outdoor spraying, and at ceiling, mid-height and floor levels for indoor house spraying. Knockdown and mortality were recorded at each checkpoint and compared by treatments.

**Results:**

Overall, Fludora Co-Max EW induced significantly higher knockdown and mortality effects in the wild insecticide-resistant *Ae. aegypti* and *Cx. quinquefasciatus* compared with K-Othrine EC. In both species, mortality rates with Fludora Co-Max EW were > 80% (up to 100%) with the ULV spray outdoors at each distance checkpoint (i.e. 10–100 m), and 100% with the ULV and TF sprays indoors at all checkpoints (i.e. ceiling, mid-height and floor). K-Othrine EC induced high mortality indoors (97.9–100%), whereas mortality outdoors rapidly declined in *Ae. aegypti* from 96.7% (10 m) to 36.7% (100 m) with the ULV spray, and from 85.0% (10 m) to 38.3% (100 m) with the TF spray. Fludora Co-Max EW spray applied as ULV spray outdoors had higher knockdown and higher killing effects on *Ae. aegypti* and *Cx. quinquefasciatus* than when applied as TF spray. Fludora Co-Max EW performed better against *Cx. quinquefasciatus* than against *Ae. aegypti*.

**Conclusions:**

Fludora Co-Max EW induced high mortality and knockdown effects against wild insecticide-resistant *Ae. aegypti* and *Cx. quinquefasciatus* Abidjan strains and performed better than K-Othrine EC. The presence of flupyradifurone and transfluthrin (with new and independent modes of action) and FFAST technology in the current Fludora Co-Max EW formulation appears to have broadened its killing capacity. Fludora Co-Max EW is thus an effective adulticide and may be a useful tool for *Aedes* and *Culex* mosquito and arbovirus control in endemic areas.

**Graphical Abstract:**

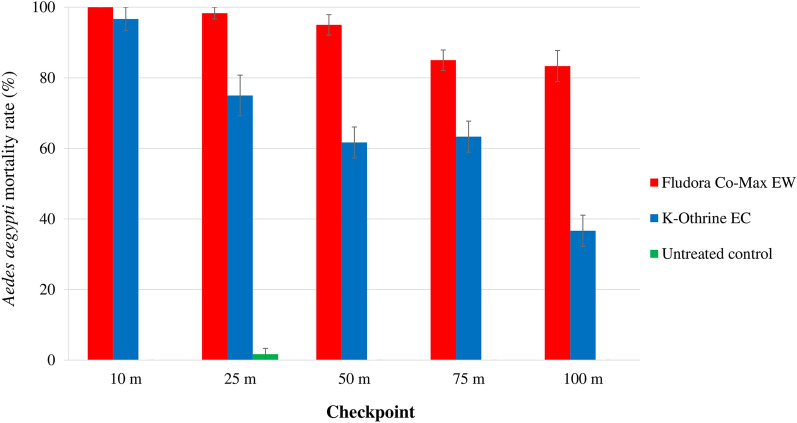

**Supplementary Information:**

The online version contains supplementary material available at 10.1186/s13071-022-05572-5.

## Background

*Aedes aegypti* and *Culex quinquefasciatus* mosquitoes are the primary vectors of arboviruses (e.g. dengue, yellow fever, chikungunya, Zika, Rift valley fever and West Nile virus) transmitted to humans and livestock worldwide [1]. It has been estimated that over 3.9 billion people are at risk of dengue fever, with about 390 million infections per year, resulting in over 100 million symptomatic cases and 10,000 deaths [2]. Originating in Africa, arboviruses and their *Aedes* vectors are now spreading rapidly across the world [3] due to accelerated urbanisation, international trade, mobility and climate change [4, 5]. Across Africa, arboviruses are a major public health threat to more than 831 million people, which is 70% of the continent’s population [6–8]. Among domestic animals, Rift Valley fever infections have caused an increased number of abortions, stillbirths and mortality, resulting in considerable economic losses [9]. Because there are no vaccines and no specific drugs against most of these arboviruses, prevention is mainly based on effective surveillance and vector control measures [1]. Space spraying of insecticides is still an important means of controlling *Aedes* and *Culex* mosquitoes and preventing arboviral outbreaks [10].

In Côte d’Ivoire, as in many African countries, dengue has become a major public health concern, with the emergence of multiple outbreaks often coupled with yellow fever co-infections [11, 12]. The majority (> 80%) of these outbreaks occurred in the city of Abidjan (approx. 6 million inhabitants) [11, 12]. The outbreak responses, conducted by the national arbovirus control programme of the Ministry of Health (MoH) and based on space sprays of insecticides (e.g. deltamethrin), have often had limited impacts on *Aedes* vector control due to insecticide resistance: *Ae. aegypti* populations were found to have recovered quickly and arboviruses to have re-emerged massively once the campaigns were over [11, 12]. Livestock were infected by Rift Valley fever virus in Côte d’Ivoire [3]. Indeed, the local *Ae. aegypti* and *Cx. quinquefasciatus* populations are both highly abundant [14, 15] and resistant to many of the insecticides (e.g. deltamethrin, permethrin, lamdacyhalothrin, propoxur and fenitrothion) used for their control [16–18].

Given the present spread of arboviruses and insecticide resistance in *Aedes* arbovirus vectors worldwide [19–21] and specifically in West Africa [22–24], there is a pressing need to develop effective vector control tools and strategies to prevent arboviral epidemics. Sustainable and rational deployment of an effective tool for outdoor and indoor vector control within insecticide resistance management programmes can reduce local vector densities. It is therefore important to develop new vector control tools that incorporate a novel insecticide molecule or a combination of insecticide molecules with different modes of action, as part of insecticide resistance management. The candidate product to be evaluated in the current study is Fludora Co-Max EW, a new mixture of formulations, developed by Bayer AG (Leverkusen, Germany) for outdoor and indoor space spraying. The Fludora Co-Max EW formulation is a combination of two insecticides, flupyradifurone and transfluthrin, and is primarily based on water emulsion with built-in anti-evaporant technology, i.e. film forming aqueous spray technology (FFAST). FFAST (e.g. as in Aqua-K-Othrine) forms a protective skin around the droplets that slows down evaporation events and ensures that the optimal droplet size is maintained longer, thereby prolonging maximal efficacy duration for longer space impaction efficiency [25].

The aim of the small-scale field study reported here was to evaluate the space spray efficacy of Fludora Co-Max EW against adult females of natural and insecticide-resistant populations of *Ae. aegypti* and *Cx. quinquefasciatus* in comparison with that of K-Othrine EC (Bayer AG; formulation containing only deltamethrin) in Abidjan, Côte d’Ivoire. As Fludora Co-Max EW is built on a combination of two new insecticides with unilateral modes of action and FFAST technology, it is expected to kill the wild pyrethroid-resistant *Ae. aegypti* and *Cx. quinquefasciatus* Abidjan strains. The results demonstrated the potential of Fludora Co-Max EW to contribute to the management of insecticide resistance in *Aedes* and *Culex* mosquitoes for the control of arboviral diseases.

## Methods

### Mosquitoes

*Aedes aegypti* and *Cx. quinquefasciatus* mosquito strains from Abidjan, Côte d’Ivoire (Abidjan strains) were used in this trial. Both strains have shown varied resistance, ranging from probable resistance to confirmed resistance, against the insecticides usually used for their control in Côte d’Ivoire, such as, for example, K-Othrine [16–18]. Recent studies detected three knockdown resistance (*kdr*) mutations (V410L, V1016I and F1534C) in the wild *Ae. aegypti* Abidjan populations, with low frequencies for the Leu 410 (0.28) and Ile1016 (0.32) alleles and high frequency for the Cys1534 allele (0.96) [18]. *Aedes* and *Culex* mosquito immatures (i.e. larvae and pupae) were collected in their common breeding sites (e.g. tyres, tins cans, discarded containers, puddles, gutters, etc.) in Abidjan (5°20′11″ N, 4°01′36″ W). The collected mosquito immatures were transported by air-conditioned car to our insectary in Agboville (100 km from Abidjan), Côte d’Ivoire for rearing under standard laboratory conditions (27 ± 2 °C; 80 ± 10% relative humidity [RH]; 12:12 h, light:dark) until the adult stage and subsequent testing. Emerged *Ae. aegypti* and *Cx. quinquefasciatus* adults (F_0_ generation) were fed on cotton wool soaked in a 10% sugar solution and maintained under the same laboratory conditions as described. Unfed females of each species, aged 3–5 days, were placed in cages at a density of 20 specimens per evaluation cage for the trials.

### Insecticide formulations and dilution

The candidate insecticide formulation Fludora Co-Max EW 78.8 (referred to hereafter as Fludora Co-Max EW) and the local standard product/insecticide K-Othrine EC 25 (referred to hereafter as K-Othrine EC), both water-based space spray concentrates, are specifically designed for dilution in water only. Fludora Co-Max EW is a combination of two insecticides, flupyradifurone (26.3 g/l) and transfluthrin (52.5 g/l) with unrelated modes of action that is combined with FFAST technology for longer space impaction efficiency; in contrast, the K-Othrine EC insecticide formulation contains only deltamethrin (25 g/l).

The trial consisted of three study arms per application method: (i) the candidate product (Fludora Co-Max EW); (ii) the positive reference control (K-Othrine EC); and (iii) the negative control (water without insecticide). The application methods were: (i) ultra-low volume cold fogging (ULV); and (ii) thermal fogging (TF), both outdoors and indoors. Each insecticide formulation was diluted with water. Fludora Co-Max EW dilution rates were provided by the manufacturer (Bayer AG) and were 1:10 (1 ml of Fludora Co-Max EW + 9 ml of water) and, 1:50 for outdoor/indoor ULV, and 1:100 and 1:100 for outdoor/indoor TF, respectively. The K-Othrine EC dilution was based on its standard dilution rates applied for mosquito control; these were 1:25 (1 ml K-Othrine EC + 24 ml of water) for outdoor/indoor ULV and 1:125 (1 ml K-Othrine EC + 124 ml of water) for outdoor/indoor TF. The insecticide products were diluted with the suitable volume of water according to the equipment manufacturer’s recommendations for the output rates required for each spraying method (Additional file 1: Table S1; Additional file 2: Fig. S1). As three replicates were performed per concentration and per insecticide formulation (including the negative control), the dilution quantities required for immediate usage were freshly prepared for each new replicate.

### Spray equipment

For outdoor application, two types of vehicle-mounted sprayers to distribute the insecticide formulations: the Micronair AU9000 cold fogging machine (Micron Group, Herefordshire, UK) for ULV spraying and the Swingfog SN 101 Pump (Swingtec GmbH, Isny, Germany) for TF spraying. For indoor application, two types of hand-held sprayers were used: the Vectorfog C150+ cold fogger (VectorFog, East Windsor, NY, USA) for ULV spraying and the Swingfog SN 50 (Swingtec GmbH) for TF spraying. As standard practice, all application equipment was calibrated to determine the output rates and actual volumes required in order to reach the product target application rates (Additional file 3: Table S2). For Fuldora Co-Max EW, the flow rates were approximately 0.98, 1.91, 0.83 and 0.17 l/min with the Micronair AU9000 fogging machine (outdoor ULV), Swingfog SN 101 Pump (outdoor TF), Vectorfog C150 + (indoor ULV) and Swingfog SN 50, respectively. For K-Othrine EC, the respective flow rates were estimated at 0.96, 1.95, 0.81 and 0.16 l/min with Micronair AU9000 (outdoor ULV), Swingfog SN 101 Pump (outdoor TF), Vectorfog C150 + (indoor ULV) and Swingfog SN 50, respectively. As no coated slides or droplet catching machines were available, the manufacturers’ guidelines for machine calibration were followed to take into account the droplet size. The nozzle for the correct volume medium diameter (VMD) size for the space spray trial was expected to be between 25 and 30 µm. The discharge rate was pre-calibrated in a separate spraying exercise. An experienced operator conducted the fogging equipment, moving along the spray line at a standard pace.

### Trial area and procedures

This phase II semi-field study was carried out using four space spray trial types: outdoor ULV, outdoor TF, indoor ULV and indoor TF (Additional file 4: Fig. S2). All trials were carried out in Agboville (5°55′41″ N, 4°13′01″ W). Outdoor trials were conducted in an open area with no vegetation, named “Place Bédié” (250 × 150 m). Indoor trials were performed in an empty room of a house, with an approximate volume of 126 m^3^ (L × W × H: 8.5 × 5.3 × 2.8 m).

The trials were designed and carried out according to the current standard WHO guidelines for outdoor and indoor space spray applications of insecticides for the control of vectors and public health pests [26, 27], as follows.

#### Outdoor fogging

A 200-m-long straight transect was designated on one side of the “Place Bédié” as a spray line to be followed by the spraying vehicle. Perpendicularly to and downwind from the spray line, 1.5-m-high poles were positioned at five distance checkpoints (10, 25, 50, 75 and 100 m) (Additional file 5: Fig. S3). A checkpoint evaluation cage with 20 mosquitoes was placed at the top of each pole. The cages were cylindrical and constructed of fine mesh fabric (nylon) with wire frame support (10 cm [diameter] × 15 cm [H] × 10 cm [tapping cover]) (Additional file 6: Fig. S4). The mesh size was approximately 1.2 mm, to allow spray droplets to pass through. The vehicle-mounted spray machine was used for the tests; for ULV and TF spraying, the vehicle speed was 12 and 10 km/h, respectively. The wind direction should be perpendicular to the vehicle travel line, and was assessed using a long low-weight tissue (e.g. toilet paper). After spraying, tested mosquitoes were left in the test area for 15-min exposure. Spraying was conducted in the morning before sunrise and in the evening after sunset. The ambient temperature and RH were monitored and recorded before and after each trial (Additional file 7: Table S3). The meteorological conditions were optimal for the space spraying, with the temperature ranging from 25 °C to 32 °C and the RH ranging from to 50% to 78%.

#### Indoor fogging

The room selected for indoor spraying was ventilated and adequately decontaminated. A total of 10 checkpoint evaluation cages per mosquito species (20 mosquitoes/cage) were placed in each room: four cages 25 cm from each corner at the ceiling and floor levels, respectively, and two at mid-height (approximatively 1.5 m from the centre of the room) (Additional file 8: Fig. S5). The windows were closed prior to spraying. An experienced technician delivered the spray either from the front door (i.e. inside-out treatment) or through an open window, with the hand-held sprayer nozzle directed towards the centre of the room to achieve adequate dispersal of the insecticide droplets. After spraying, all doors and windows opening onto the outdoors were closed during the 20 min that the mosquitoes were exposed to the insecticide. The environmental conditions in the test room were recorded before and after each trial (Additional file 7: Table S3). The weather was fair during the test; the air temperature ranged from 26 °C to 33 °C, and the RH varied from to 50% and 79%.

#### Evaluation

After the specified exposure period, knockdown was immediately recorded at the time of collection. Tested mosquitoes were then rapidly transferred into clean holding containers (plastic cups) covered with mesh cloth using an appropriate aspirator and brought back to the field station. Mosquitoes knocked out were recorded every 3 and 2 min alternatively during a 60-min period, thus enabling knockdown to be recorded at 0, 10, 30, 40, 50 and 60 min. The tested mosquitoes were provided with a cotton wool pad soaked in 10% sucrose and transferred to the laboratory where they were held under standard conditions (27 °C ± 2 °C; 80 ± 10% RH; 12:12 h, light:dark) for 24 h. The 24-h mortality rate was then recorded.

#### Test validity

The mortality rates in the concurrent negative control cages were < 5%. Thus, the results from the treated samples were directly accepted.

### Assessment of side effects

We assessed the possible side effects (e.g. itching, dizziness or nose running) among the sprayers and the occupants of the sprayed house through interviews.

### Statistical analysis

Raw data were double entered into Excel (Microsoft Corp., Redmond, WA, USA) using double key entry and the data checked for outliers. The knockdown rate was expressed as the percentage of specimens knocked out (i.e. numerator) among the number of mosquitoes tested (i.e. denominator). The mortality rate was calculated as the percentage of dead specimens (i.e. numerator) among mosquitoes exposed (i.e. denominator) to treatment for each insecticide dose. The mortality rates (mean ± standard error) were compared using a one-way analysis of variance, followed by Bonferroni’s correction. The data were analysed by the Kruskal–Wallis test only when they showed significant deviations from normality (i.e. when the Shapiro–Wilk W-test for normal data was significant). A significance level of 5% was set for statistical testing. All statistical analyses were conducted using Stata version 16.0 (Stata Corp., College Station, TX, USA).

## Results

### Outdoor ULV efficacy

#### Knockdown

At 0 min post-exposure, the knockdown rates for the Fludora Co-Max EW and K-Othrine EC ULV space sprays outdoors at the checkpoint distances (10–100 m) ranged from 100.0% to 85.0% and from 83.3% to 28.3%, respectively, in *Ae. aegypti*, and from 100.0% to 3.3% and from 96.7% to 38.3%, respectively, in *Cx. quinquefasciatus* (Additional file 9: Table S4). At 60 min post-exposure, Fludora Co-Max EW and K-Othrine EC induced respective knockdown rates that varied from 100.0% to 95.0% and 91.7% to 30.0% in *Ae. aegypti*, and from 100.0% to 95.0% and from 100.0% to 78.3% in *Cx. quinquefasciatus*, at distances ranging from 10 to 100 m. Thus, in both mosquito species, Fludora Co-Max EW effected faster and higher knockdown rates at longer distances compared to K-Othrine EC.

#### Mortality

Outdoor ULV space sprays of Fludora Co-Max EW and K-Othrine EC resulted in overall mortality rates of 92.3% ± 2.1% and 66.7% ± 5.5%, respectively, in *Ae. aegypti*, and of 99.7% ± 0.3% and 95.3% ± 1.6%, respectively, in *Cx. quinquefasciatus* (Additional file 10: Table S5). Fludora Co-Max EW mortality rates were significantly higher compared with K-Othrine EC in *Ae. aegypti* (*χ*^2^ = 11.13; *df* = 1; *P* < 0.05) and *Cx. quinquefasciatus* (*χ*^2^ = 6.30; *df* = 1; *P* < 0.05). Mortality rates were very high with Fludora Co-Max EW, being > 90% in both mosquito species; in comparison, mortality rates with K-Othrine EC was > 90% only in *Cx. quinquefasciatus.*

**Fig. 1:**
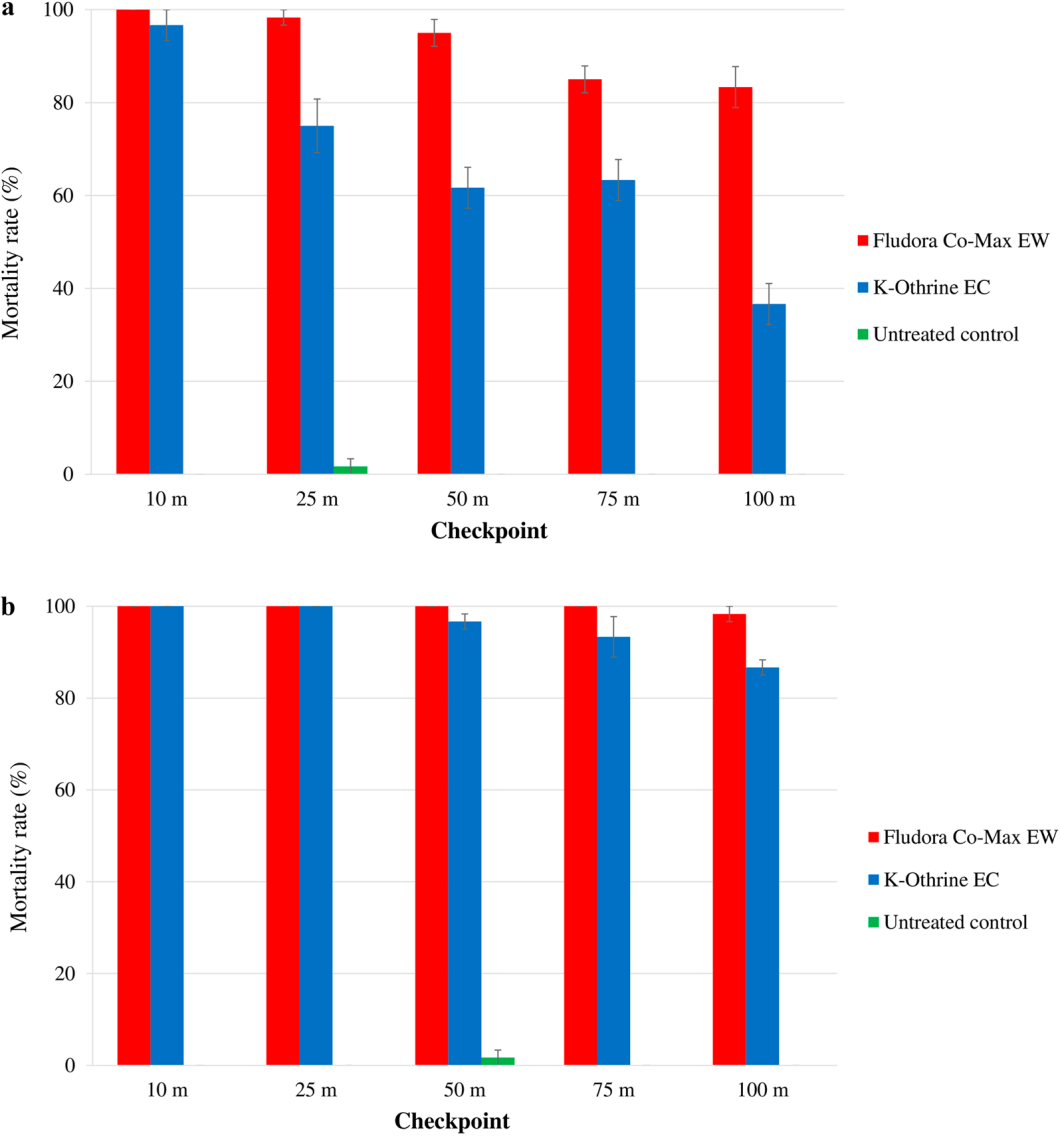
Mortality of the wild insecticide-resistant *Aedes aegypti* and *Culex quinquefasciatus* Abidjan strain mosquitoes exposed to outdoor ultra-low volume (ULV) space sprays of Fludora Co-Max EW and K-Othrine EC. **a** *Ae. aegypti*, **b** *Cx. quinquefasciatus*. Error bars show the standard error of the mean (SEM)

The mortality rates for the two mosquito species varied substantially depending on the distance checkpoint (Fig. 1).

##### *Aedes aegypti:*

Fludora Co-Max EW ULV space spray induced high mortality rates in *Ae. aegypti* that varied within a narrow range: 100.0% ± 0.0% at 10 m to 83.3% ± 4.4% at 100 m. Only the mortality rate at 100 m was significantly lower than those at the other distance checkpoints (*F* = 56.00 ; *df* = 1; *P* < 0.05, *type of test* and the *degree of freedom (df)*). Conversely, K-Othrine EC ULV induced a high mortality rate of 96.7% ± 3.3% at 10 m that rapidly declined to 36.7% ± 4.4% at 100 m, with the mortality rates at the checkpoints from 50 to 100 m being significantly lower than those at the 10 and 25 m checkpoints (all *F* = 7.61, *df* = 4; *P* < 0.05). Between 25 and 100 m, Fludora Co-Max EW induced significantly higher mortality rates than K-Othrine EC at each distance checkpoint (*F* = 23.18; *df* = 4; *P* < 0.001). The mortality rates associated with Fludora Co-Max EW spray were always in excess of 80%, exceeding 90% between the 10 m (100.0% ± 0.0%) and 25 m (91.7% ± 7.6%) checkpoints while the mortality rates associated with K-Othrine EC spray were < 80% between the 25 and 100 m checkpoints.

##### *Culex quinquefasciatus:*

The mortality rates in *Cx. quinquefasciatus* exposed to outdoor ULV space spray ranged from 100.0% ± 0.0% at 10 m to 98.3% ± 1.7% at 100 m for Fludora Co-Max EW, and from 100.0% ± 0.0% at 10 m to 86.7% ± 1.7% at 100 m for K-Othrine EC. While there was no significant reduction in the mortality rate among *Cx. quinquefasciatus* mosquitoes exposed to Fludora Co-Max EW mortality from the 10 to 100 m checkpoints (*χ*^2^ = 4.00; *df* = 4; *P* = 0.406), mortality among the mosquitoes exposed to K-Othrine EC significantly decreased from 10 to 100 m (*χ*^2^ = 9.91; *df* = 4; *P* < 0.05). At 100 m, the mortality rates were significantly higher in mosquitoes exposed to Fludora Co-Max EW (98.3% ± 1.7%) than in those exposed to K-Othrine EC (86.7% ± 1.7%) (*χ*^2^ = 4.09; *df* = 1; *P* < 0.05). Overall, the mortality rates were > 90% for Fludora Co-Max EW at any distance checkpoint, while K-Othrine EC mortality was < 90% at the 100 m checkpoint.

### Outdoor TF efficacy

#### Knockdown

At 0 min post-exposure, Fludora Co-Max EW and K-Othrine EC outdoor TF space sprays provided knockdown rates estimated at 95.0–71.0% and 71.7–36.7%, respectively, in *Ae. aegypti*, and at 98.3–28.3% and 66.7–16.7%, respectively, in *Cx. quinquefasciatus* from 10 to 100 m (Additional file 11: Table S6). At 60 min post-exposure, the respective knockdown rates of Fludora Co-Max EW and K-Othrine EC were estimated at 100.0–85.0% and 85.0–36.7%, respectively, in *Ae. aegypti*, and at 100.0–51.7% and 83.3–63.3%, respectively, in *Cx. quinquefasciatus*. Fludora Co-Max EW thus caused more rapid and higher knockdown effects and for a longer distance compared with K-Othrine EC.

#### Mortality

With outdoor TF space sprays, the Fludora Co-Max EW and K-Othrine EC overall mortality rates were 73.3% ± 5.5% and 57.0% ± 5.2%, respectively, in *Ae. aegypti*, and 96.3% ± 2.1% and 86.3% ± 3.1%, respectively, in *Cx. quinquefasciatus* (Additional file 12: Table S7). Fludora Co-Max EW induced statistically higher overall mortality rates compared with K-Othrine EC in *Ae. aegypti* (*F* = 4.62; *df* = 1; *P* < 0.05) and in *Cx. quinquefasciatus* (*χ*^2^ = 7.32; *df* = 1; *P* < 0.05). Only in *Cx. quinquefasciatus* was the Fludora Co-Max EW mortality rate > 90%.

**Fig. 2:**
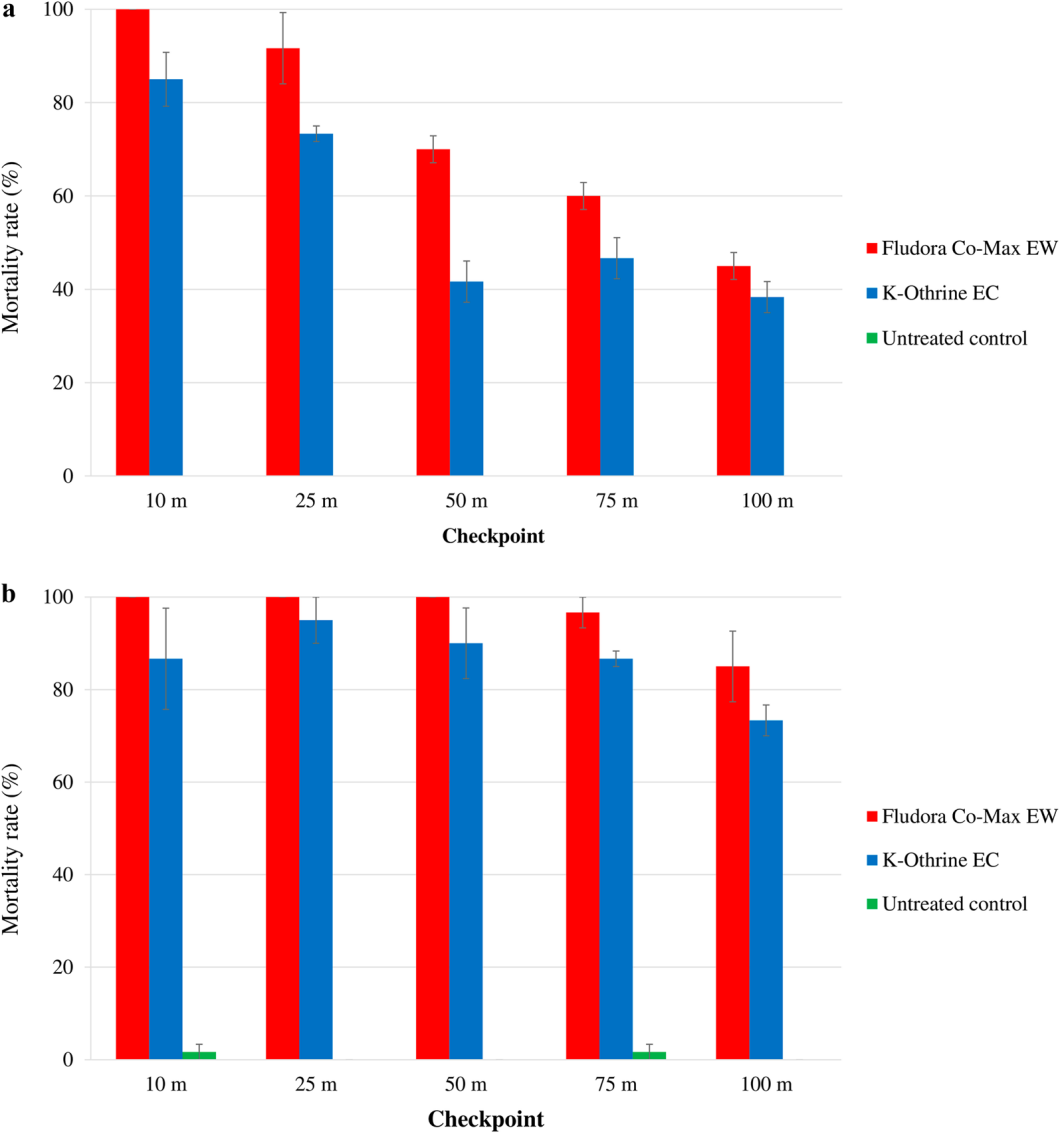
Mortality of the wild insecticide-resistant *Ae. aegypti* and *Cx. quinquefasciatus* Abidjan strain mosquitoes exposed to outdoor thermal fogging (TF) space sprays of Fludora Co-Max EW and K-Othrine EC. **a** *Ae. aegypti*, **b** *Cx. quinquefasciatus*. Error bars show the SEM

The mortality rates varied significantly among the distance checkpoints (Fig. 2).

##### *Aedes aegypti:*

Outdoor TF space sprays of Fludora Co-Max EW induced high mortality rates in *Ae. aegypti* of 100.0% ± 0.0% at 10 m and 91.7% ± 7.6% at 25 m; the mortality rate significantly decreased from 70.0% ± 2.9% at 50 m to 45.0% ± 2.9% at 100 m (*F* = 57.34; *df* = 4; *P* < 0.001). The mortality rates in mosquitoes exposed to K-Othrine EC declined suddenly from 85.0% ± 5.8% at 10 m to 38.3% ± 3.3% at 100 m, with significant differences between the distance checkpoints (*F* = 25.27; *df* = 4; *P* < 0.001). The Fludora Co-Max EW outdoor TF spray caused higher mortality than the K-Othrine EC outdoor TF spray, with significant differences at 25 and 50 m (all *F *= 4.62; *df* = 1; *P* < 0.05), but without statistical differences at 10, 75 and 100 m (all *F *= 6.75; *df* = 1; *P* = 0.0602). All mortality rates due to Fludora Co-Max EW were > 90% at 10 and 25 m, whereas those due to K-Othrine were < 90% at all checkpoints.

##### *Culex quinquefasciatus:*

Outdoor TF space sprays of Fludora Co-Max EW and K-Othrine EC induced high mortality in *Cx. quinquefasciatus* mosquitoes. The mortality rates decreased, without significant differences, from 100% ± 0.0% at 10 m and 85.0% ± 7.6% at 100 m for Fludora Co-Max EW (*χ*^2^ = 6.71; *df* = 4; *P* = 0.1520), and from 95.0% ± 5.0% at 25 m to 73.3% ± 3.3% at 100 m for K-Othrine EC (*F* = 1.49; *df* = 4; *P* = 0.2776). The mortality rates were substantially higher in *Cx. quinquefasciatus* mosquitoes exposed to Fludora Co-Max EW compared with those exposed to K-Othrine EC, but the difference lacked statistical difference (*χ*^2^ = 16.71; *df* = 9; *P* = 0.0535). Mortality rates were > 90% for both Fludora Co-Max EW and K-Othrine EC up to the 100 m and 75 m checkpoints, respectively.

### Indoor ULV efficacy

#### Knockdown

At 0 min after exposure, indoor ULV space sprays of Fludora Co-Max EW and K-Othrine EC induced knockdown rates of 99.6–100.0% and 89.2–93.3%, respectively, in *Ae. aegypti*, and of 92.1–100.0% and 75.0–92.1%, respectively, in *Cx. quinquefasciatus* (Additional file 13: Table S8). At 60 min post-exposure, Fludora Co-Max EW and K-Othrine EC knockdown rates were estimated at 100.0% and 97.5–99.2%, respectively, for *Ae. aegypti*, and 99.2–100.0% and 97.5–100.0%, respectively, for *Cx. quinquefasciatus*. Thus, knockdown effects were higher and faster with Fludora Co-Max EW than with K-Othrine EC.

#### Mortality

Indoor UVL space sprays of Fludora Co-Max EW and K-Othrine EC resulted in overall high mortality rates of 100.0% ± 0.0% and 97.8% ± 0.6%, respectively, in *Ae. aegypti* (Additional file 14: Table S9). Fludora Co-Max EW induced statistically higher mortality rates compared with K-Othrine EC (*χ*^2^ = 4.918; *df* = 1; *P* < 0.05). Both insecticide products induced overall mortality rates of 100.0% ± 0.0% in *Cx. quinquefasciatus*. Therefore, the mortalities with both Fludora Co-Max EW and K-Othrine EC were largely > 90% in both mosquito species.

**Fig. 3:**
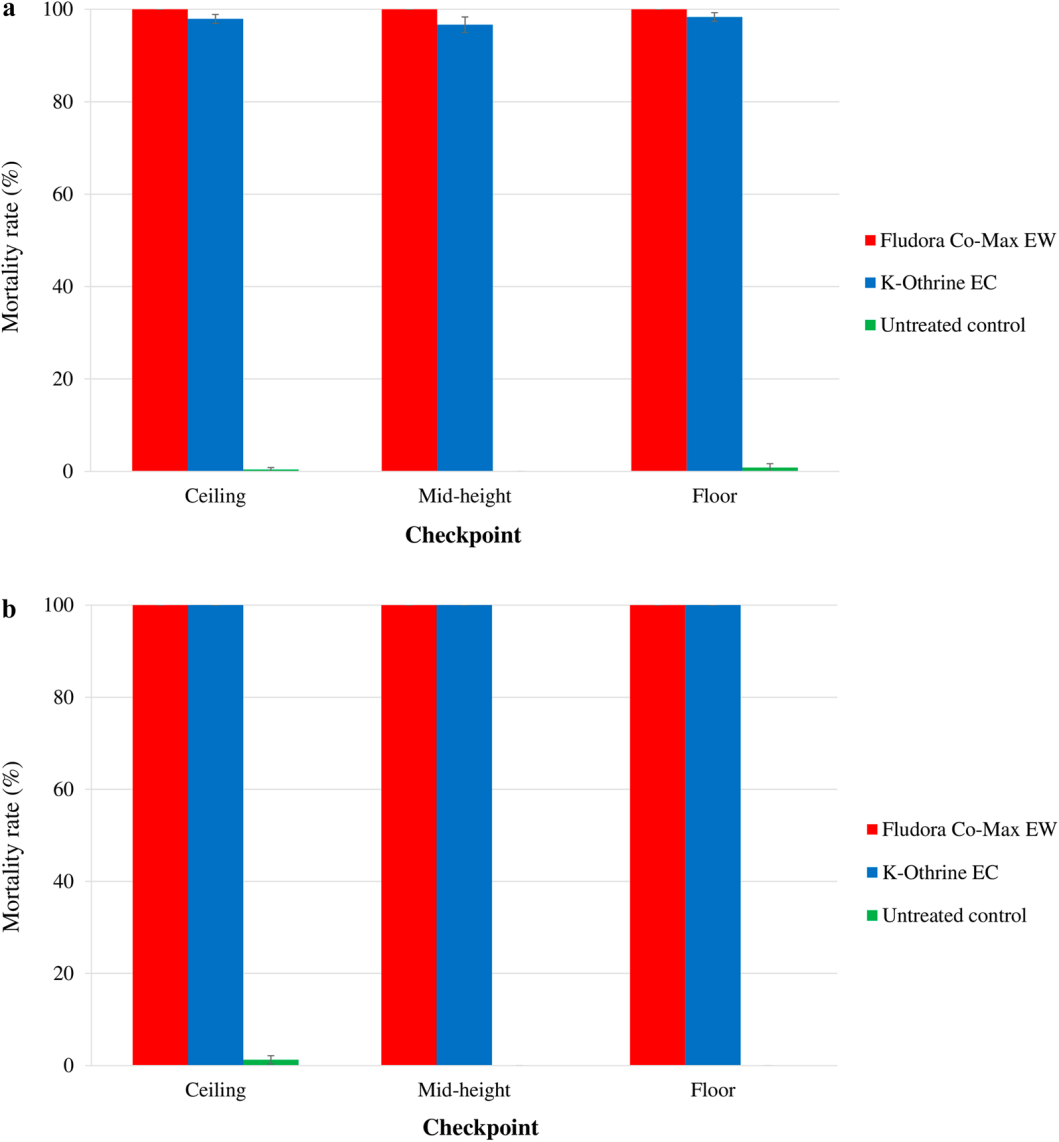
Mortality of the wild insecticide-resistant *Ae. aegypti* and *Cx. quinquefasciatus* Abidjan strain mosquitoes exposed to indoor TF space sprays of Fludora Co-Max EW and K-Othrine EC. **a** *Ae. aegypti*, **b** *Cx. quinquefasciatus*. Error bars show the SEM

The mortality rates were very high at all position level checkpoints, but varied significantly among the checkpoints (Fig. 3).

##### *Aedes aegypti:*

In* Ae. aegypti*, Fludora Co-Max EW induced 100.0% ± 0.0% mortality at all of the cage level position checkpoints. K-Othrine EC mortality rates were also high and varied from 98.3% ± 0.9% at the floor level to 96.7% ± 1.7% at the mid-height level. Compared with K-Othrine EC, Fludora Co-Max EW mortality rates were statistically higher at the ceiling (*χ*^2^ = 4.57; *df* = 1; *P* < 0.05), but not significantly different at the mid-height level (*χ2* = 4.57; *df* = 1; *P* = 0.0578) and floor (*χ*^2^ = 3.27; *df* = 1; *P* = 0.0704).

##### *Culex quinquefasciatus:*

In* Cx. quinquefasciatus*, both Fludora Co-Max EW and K-Othrine EC produced very high mortality rates of 100.0% ± 0.0% at all position level checkpoints in the house.

### Indoor TF efficacy

#### Knockdown

At 0 min after exposure, Fludora Co-Max EW and K-Othrine EC indoor TF space spraying induced high knockdown effects, with respective knockdown rates of 100.0% and 89.2–92.1% in *Ae. aegypti*, and 99.2–100.0% and 78.3–95.8% in *Cx. quinquefasciatus* (Additional file 15: Table S10). At 60 min post-exposure, Fludora Co-Max EW and K-Othrine EC knockdown rates were, respectively, 100.0% and 98.8–100.0% for *Ae. aegypti,* and 100.0% and 93.8–100.0% for *Cx. quinquefasciatus*. Overall, the knockdown effects were faster and higher with Fludora Co-Max EW than with K-Othrine EC.

#### Mortality

With indoor TF space sprays, overall mortality rates in *Ae. aegypti* were 100.0% ± 0.0% for Fludora Co-Max EW and 99.3% ± 0.3% for K-Othrine EC (Additional file 16: Table S11). Thus, Fludora Co-Max EW performed significantly better than K-Othrine EC (*χ*^2^ = 4.21; *df* = 1; *P* < 0.05). In *Cx. quinquefasciatus*, both Fludora Co-Max EW and K-Othrine EC provided very high mortality rates of 100.0% ± 0.0%. The overall mortality rates with both insecticide products were > 90% in the two mosquito species.

**Fig. 4:**
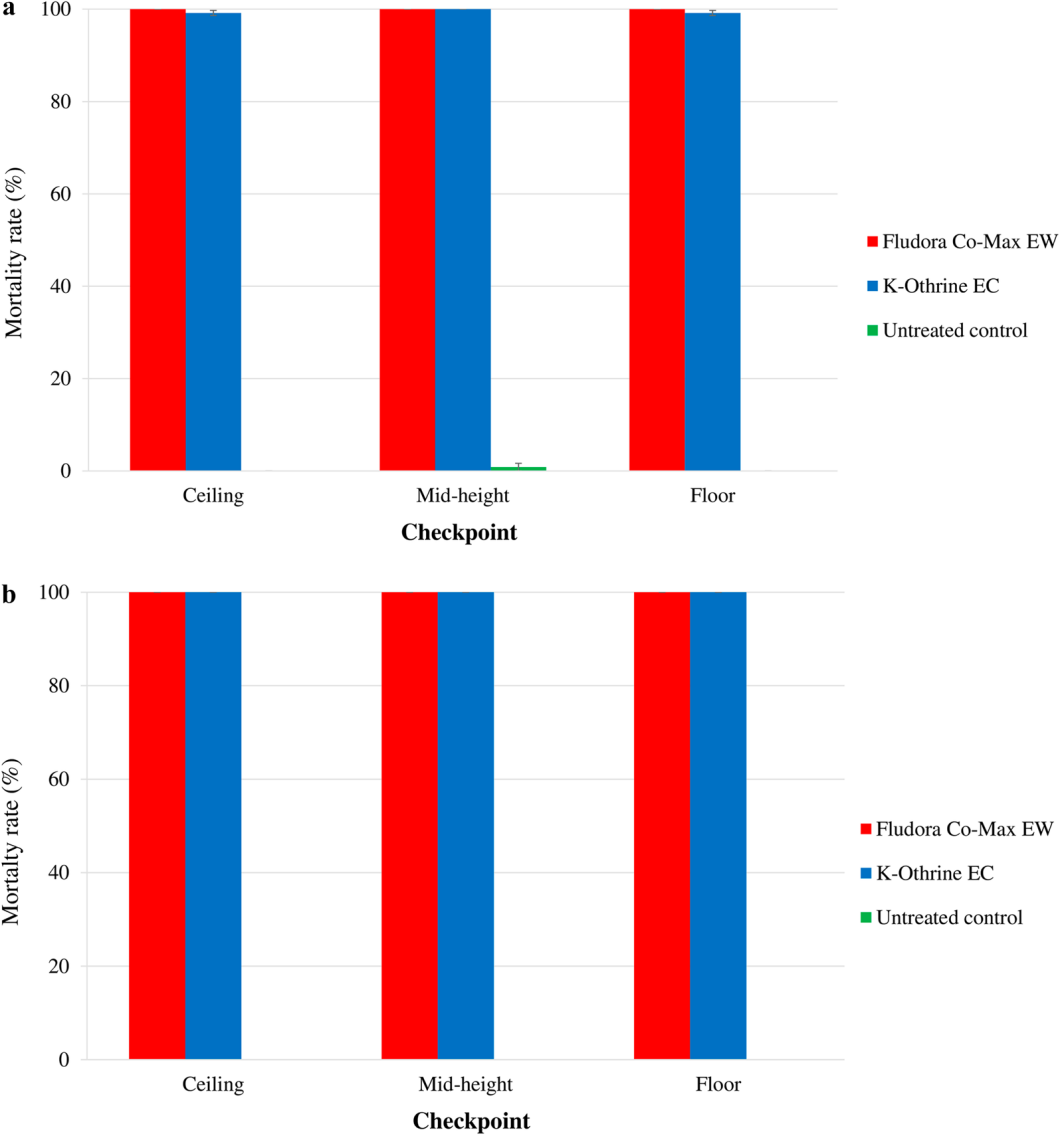
Mortality of the wild insecticide-resistant *Ae. aegypti* and *Cx. quinquefasciatus* Abidjan strain mosquitoes exposed to indoor TF space sprays of Fludora Co-Max EW and K-Othrine EC. **a** *Ae. aegypti*, **b** *Cx. quinquefasciatus*. Error bars show the SEM

The mortality rates varied significantly among the position level checkpoints (Fig. 4).

##### *Aedes aegypti:*

In *Ae. aegypti*, Fludora Co-Max EW always induced a 100.0% mortality rate at any of the level checkpoints, while K-Othrine EC caused high mortality rates that varied from 99.2% ± 0.6% at the ceiling and the floor to 100.0% ± 0.0% at the mid-height level. However, there was no significant difference in mortality rate due to K-Othrine EC between the level checkpoints (*χ*^2^ = 1.12; *df* = 1; *P* = 0.5725). No significant differences were observed between Fludora Co-Max EW and K-Othrine EC mortality rates regardless of the level of the checkpoints in the house (*χ*^2^ = 0.787; *df* = 1; *P* = 0.3750). However, Fludora Co-Max EW performed better than K-Othrine EC.

##### *Culex quinquefasciatus:*

For *Cx. quinquefasciatus*, both Fludora Co-Max EW and K-Othrine EC products, regardless of application method, scored mortality rates of 100.0% ± 0.0% at all position checkpoints.

### Fludora Co-Max EW space spray efficacy: ULV versus TF

**Fig. 5:**
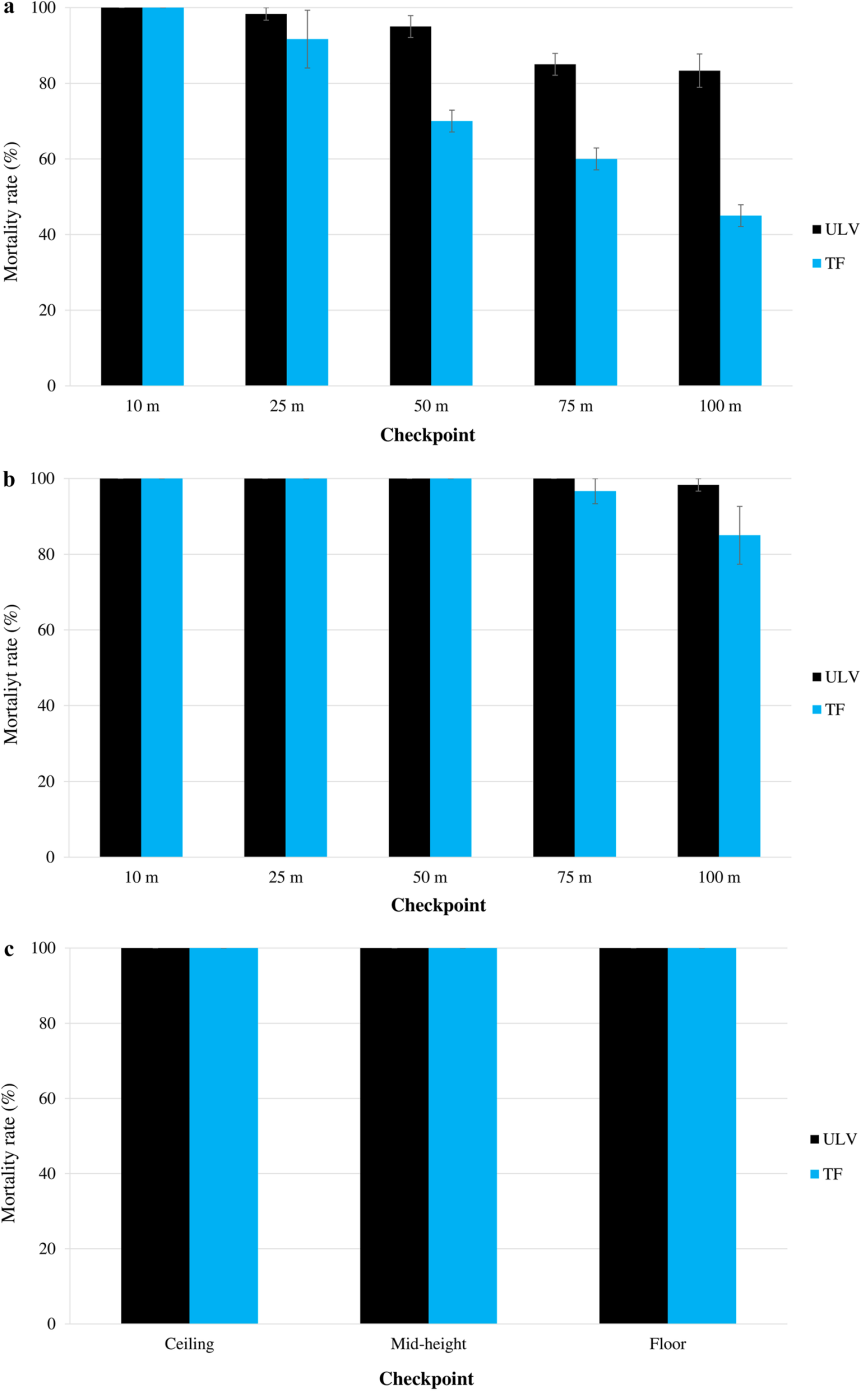

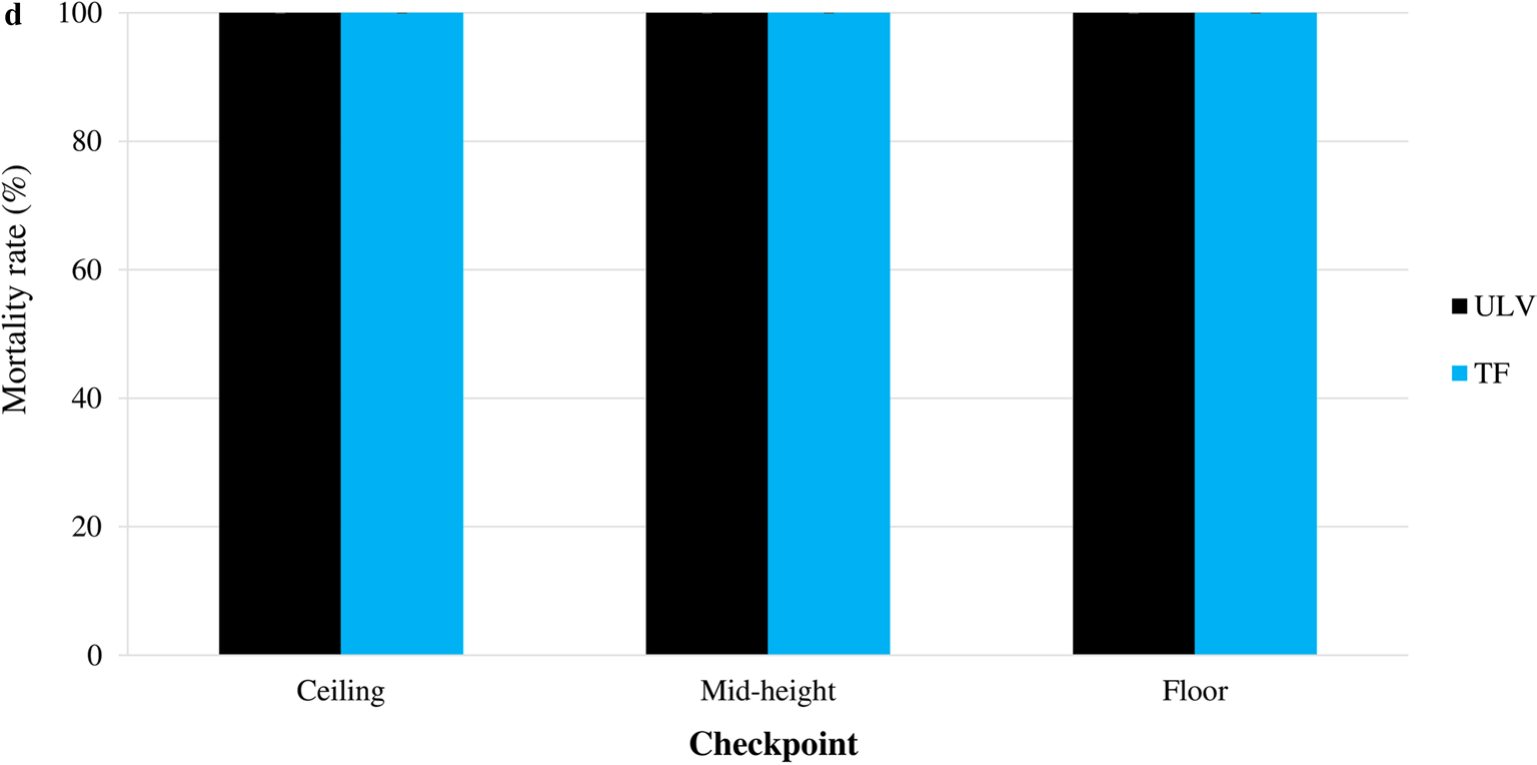
Comparison between ULV and TF space spray applications of Fludora Co-Max EW against the wild insecticide-resistant *Ae. aegypti* and *Cx. quinquefasciatus* Abidjan strain mosquitoes. **a** Outdoor ULV, **b** outdoor TF, **c** indoor ULV, **d** indoor TF. Error bars show the SEM

For outdoor space sprays, the overall mortality rates with Fludora Co-Max EW were 92.3% ± 2.1% with ULV and 73.3% ± 5.5% with TF in *Ae. aegypti*, and 99.7% ± 0.3% with ULV and 96.3% ± 2.2% with TF in *Cx. quinquefasciatus*. Compared with TF, ULV showed higher mortality rates, and the difference was significant for *Ae. aegypti* (*χ*^2^ = 5.52; *df* = 1; *P* < 0.05), but not for *Cx. quinquefasciatus* (*χ*^2^ = 1.34; *df* = 1; *P* = 0.2469). The mortality rates were especially high in *Ae. aegypti*, varying from 100.0% ± 0.0% at 10 m to 83.3% ± 4.4% at 100 m for ULV; in comparison, there was a marked decline in mortality from 100.0% ± 0.0% at 10 m to 45.0% ± 2.9% at 100 m when using TF (Fig. 5). *Aedes aegypti* mortality rates were statistically higher with ULV than with TF at 50, 75 and 100 m (*F* = 33.32; *df* = 5; *P* < 0.001).

For Fludora Co-Max EW indoor space sprays, ULV and TF both induced 100.0% ± 0.0% mortality in both *Ae. aegypti* and *Cx. quinquefasciatus* overall and at all level checkpoints in the house (Fig. 5). Thus, indoor, no differences were found between ULV and TF mortality rates in either *Ae. aegypti* and or *Cx. quinquefasciatus*.

#### Perceived side effects

No negative side effects were reported among the sprayers, the operators and the occupants of the sprayed house during and after spraying.

## Discussion

The current phase II study aimed to evaluate and compare the outdoor and indoor space spray efficacy of Fludora Co-Max EW (mixture of transfluthrin and flupyradifurone with FFAST technology) with K-Othrine EC (deltamethrin-only product) against caged adult *Ae. aegypti* and *Cx. quinquefasciatus* mosquitoes from Abidjan, Côte d’Ivoire, using ULV and TF spraying. To our knowledge, this study represents one of the first small-scale trials evaluating the space spray efficacy of an insecticide against *Aedes* and *Culex* mosquitoes in sub-Saharan Africa. The results revealed that Fludora Co-Max EW was effective against wild insecticide-resistant *Ae. aegypti* and *Cx. quinquefasciatus*, and performed better than K-Othrine EC in the field.

Overall, the current semi-field evaluations showed that ULV and TF space spray of Fludora Co-Max EW outdoors and indoors effected high knockdown and mortality rates (> 90%) in both *Ae. aegypti* and *Cx. quinquefasciatus* Abidjan strains, and the values were higher than or equal to those with K-Othrine EC. Additionally, knockdown effects in both strains were faster with Fludora Co-Max EW than with K-Othrine EC. The reduction in susceptibility and mortality to K-Othrine EC (deltamethrin being the only active ingredient) in *Aedes* and *Culex* Abidjan strain mosquitoes may be due to their resistance to pyrethroids [16–18], as reported on the island of Martinique [28]. However, the high effectiveness of Fludora Co-Max EW against the same insecticide-resistant mosquito strains may be explained by the mixture of two active ingredients, namely transfluthrin and flupyradifurone. Indeed, the active ingrediants of the Fludora Co-Max EW formulation, the butenolide flupyradifurone and the pyrethroid transfluthrin, represent two unrelated modes of action and were, in fact, chosen for their complementary action, which makes it harder for resistance to develop in exposed mosquitoes and helps to control pyrethroid-resistant mosquitoes [29, 30]. Flupyradifurone belongs to the group of nicotinic acetylcholine receptor (nAChR) competitive modulators that bind to the acetylcholine site on nAChRs, causing a range of symptoms from hyper-excitation to lethargy and paralysis [29, 30]. Transfluthrin belongs to the group of sodium channel modulators that keep sodium channels open, causing hyper-excitation as well and, in some cases, nerve block [30]. As reported by the WHO [27], an overall adult vector mortality of at least 90% is considered to be effective. Thus, Fludora Co-Max EW can be recommended for the control of *Ae. aegypti* and *Cx. quinquefasciatus* outdoors and indoors, even in areas with high insecticide resistance. In general, mortality in both *Aedes* and *Culex* mosquitoes was higher in all indoor and outdoor cages for the ULV spraying when compared with the TF spraying, but this difference was only significant outdoors. Moreover, specific differences were found in Fludora Co-Max EW and K-Othrine EC efficacy according to the methods and the distance or position checkpoints.

The outcomes of this semi-field study demonstrated that outdoor ULV space spraying of Fludora Co-Max EW produced maximum mortality rates (100%) in *Ae. aegypti* at distances of up to 25 m, decreasing at greater distances to above 90% up to 50 m, and 80% up to 100 m. In contrast, the mortality rates due to K-Othrine EC rapidly dropped down below 80% from ≥ 25 m and continued to decrease up to 100 m. In *Cx. quinquefasciatus,* the mortality rates from Fludora Co-Max EW varied between 100% at 10 m and 98% at 100 m, while the K-Othrine EC-induced mortality rates were 100% at 10 m and decreased to 87% at 100 m. The outdoor TF space spray of Fludora Co-Max EW induced mortality rates in *Ae. aegypti* that were 90–100% between 10 and 25 m, and which significantly decreased to 70–45% from 50 to 100 m; the mortality rates in *Cx. quinquefasciatus*in decreased from 100% at 10 m to 85% at 100 m. The capacity of Fludora Co-Max EW to kill mosquitoes over a long distance in ULV spraying and the lower mortality achieved with TF (85%) compared to ULV (100%) are consistent with the results produced by the Kontrol (permethrin and piperonyl butoxide) insecticide against *Ae. aegypti* in a study Starke, Florida, USA [10]. Indeed, Fludora Co-Max EW incorporates FFAST anti-evaporant technology, and during the formation of the spray droplets, the anti-evaporant agent forms a protective skin around the droplets that slows down evaporation events. This process ensures that the optimal droplet size is maintained longer, prolonging maximal efficacy duration, possibly allowing Fludora Co-Max EW to achieve higher mortality up to 100 m. Otherwise, the reduction in Fludora Co-Max EW mortality with TF outdoors might be attributed to thermal effect that may have reduced the size of spray droplets. This may have allowed the wind to change the direction of the droplets and prevent the insecticide from reaching and killing the caged mosquitoes at the longer distance outdoors. Indeed, sudden changes of wind movement and droplet size play an important role in drifting, and may affect the homogeneity of droplet depositions [31].

The results of this study showed that indoor applications of Fludora Co-Max EW provided 100% knockdown and approximately 100% mortality in local wild insecticide-resistant populations of *Ae. aegypti* and *Cx. quinquefasciatus*, with both ULV and TF spraying. While K-Othrine EC was able to induce high knockdown (92–100%) and mortality (98–100%), the rates were lower than those induced by Fludora Co-Max EW. Moreover, Fludora Co-Max EW induced high mortality (approx. 100%) in both mosquito species at all position level checkpoints in a house, as reported for Kontrol indoor ULV spray in the USA [9]. The low impact of natural unpredictable changes in wind movement inside of the house may have greatly improved Fludora Co-Max EW UVL and TF efficacy indoors by increasing the exposure of the mosquitoes to this insecticide [10, 31, 32].

Although Fludora Co-Max EW showed overall high efficacy against *Aedes* and *Culex* mosquitoes in this study, additional investigations are needed to address the methodological limitations to gain a better understanding of the variations in its mortality effects. Indeed, due to logistical limitations, we did not collect Fludora Co-Max EW droplets to evaluate the size of droplets and deposition of active ingredients (i.e. transfluthrin and flupyradifurone). Evaluations of Fludora Co-Max EW droplet size and active ingredient deposition in ULV and TF applications outdoors and indoors are required to deepen our understanding of the variations in its efficacy. For outdoor spraying, assessment of local variations in wind direction, speed and viscosity and their impacts on Fludora Co-Max EW droplet size and deposition for each method (ULV and TF) used and at each distance checkpoint may help to improve our understanding of the effects of FFAST technology and variations of mosquito mortality along the distance from the spraying line.

Ultimately, Fludora Co-Max EW appears to be a promising tool targeting adult vectors outdoors and indoors for the prevention, control and the outbreak responses against arboviruses, even in areas with insecticide resistance. Additionally, no adverse events and complaints were reported among the sprayers and the occupants of the sprayed house during and after the spraying events during the current small-scale field trials. This absence of negative effects may increase community acceptability and adherence for the large-scale use of Fludora Co-Max EW. Such usage may, in turn, be beneficial to the optimal coverage of application required to interrupt the transmission of arboviral diseases. The use of water as a solvent may have improved the effectiveness of Fludora Co-Max EW and will reduce the environmental impact and costs of spraying [33]. Overall, ULV application should be recommended over TF for outdoor and indoor sprays, as ULV induced higher or comparable mortalities in both *Aedes* and *Culex* throughout the study compared with TF. However, further validations are needed in a large-scale trial to assess the acceptability of Fludora Co-Max EW and for the operational vector control of free-flying and indoor-resting natural populations of *Aedes* and *Culex* mosquitoes [34–37].

## Conclusions

The results of this small-scale field study demonstrated that outdoor and indoor space spraying of Fludora Co-Max EW effectively induced high knockdown (up to 100%) and mortality rates (up to 100%) in caged wild insecticide-resistant *Ae. aegypti* and *Cx. quinquefasciatus* strains from Abidjan, Côte d’Ivoire. While both ULV and TF space spray methods displayed similar efficacy indoors, ULV application was found to be more suitable in outdoor space spray situations. Overall, the knockdown and mortality effects in both mosquito species were higher with Fludora Co-Max EW, a formulation of flupyradifurone-transfluthrin mixed with FFAST technology) compared with the deltamethrin-only K-Othrine EC. The presence of flupyradifurone and transfluthrin in one formulation (with unrelated modes of action) and FFAST technology in Fludora Co-Max EW seems to have broadened its killing capacity. Fludora Co-Max EW thus appears to be an effective tool for outdoor and indoor space sprays targeting areas with insecticide-resistant *Ae. aegypti* and *Cx. quinquefasciatus* mosquitoes for arboviral prevention, control and outbreak responses.

## Supplementary Information

**Additional file 1: Table S1.** Tentative dose calculation of the insecticide product formulation tested.

**Additional file 2: Figure S1.** Dilution of Fludora Co-Max EW and K-Othrine performed in Agboville, Côte d’Ivoire.

**Additional file 3: Table S2.** Meteorological data recorded in the field during the small-scale trials for testing Fludora Co-Max EW and K-Othrine EC against *Aedes aegypti* and *Culex quinquefasciatus* in Agboville, Côte d’Ivoire.

**Additional file 4: Figure S2.** Different methods used for the semi-field evaluation of the efficacy of Fludora Co-Max EW and K-Othrine against *Aedes aegypti* and *Culex quinquefasciatus* Abidjan strain mosquitoes in Agboville, Côte d’Ivoire. **a** outdoor ULV, **b** outdoor TF, **c** indoor ULV, and **d** indoor TF. ULV, Ultra-low volume; TF, thermal fogging.

**Additional file 5: Figure S3.** Outdoor trial semi-field station with 1.5-m tall poles placed at five different distance checkpoints. 1, 10 m; 2, 25 m; 3, 50 m; 4, 75 m; 5; 100 m.

**Additional file 6: Figure S4.** Cylindrical cages constructed of fine mesh fabric (nylon) with wire frame support (diameter 10 cm × height 15 cm × tapping cover 10 cm) and containing adult mosquitoes for outdoor trial. **a** Front view, **b** profile view.

**Additional file 7: Table S3.** Parameters of calibration of the sprayers for outdoor and indoor application against *Aedes aegypti* and *Culex quinquefasciatus* in Agboville, Côte d’Ivoire.

**Additional file 8: Figure S5.** Indoor trial semi-field station with mosquito cages installed at different level checkpoints in a house. **a** Ceiling, **b** mid-height, **c** floor.

**Additional file 9: Table S4.** Knockdown rate at time intervals post-application in the wild insecticide-resistant *Aedes aegypti* and *Culex quinquefasciatus* Abidjan strain mosquitoes exposed to outdoor ULV space spray of Fludora Co-Max EW and K-Othrine EC. ULV, ultra-low volume.

**Additional file 10: Table S5.** Mortality of the wild insecticide-resistant *Aedes aegypti* and *Culex quinquefasciatus* Abidjan strain mosquitoes exposed to Fludora Co-Max EW and K-Othrine EC using outdoor ULV space spray. ULV, ultra-low volume.

**Additional file 11: Table S6.** Knockdown rate at time intervals post-application in wild insecticide-resistant *Aedes aegypti* and *Culex quinquefasciatus* Abidjan strain mosquitoes exposed to outdoor TF space spray of Fludora Co-Max EW and K-Othrine EC. TF, thermal fogging.

**Additional file 12: Table S7.** Mortality of the wild insecticide-resistant *Aedes aegypti* and *Culex quinquefasciatus* Abidjan strain mosquitoes exposed to Fludora Co-Max EW and K-Othrine EC using outdoor TF space spray.

**Additional file 13: Table S8.** Knockdown rate at time intervals post-application in wild insecticide-resistant *Aedes aegypti* and *Culex quinquefasciatus* Abidjan strain mosquitoes exposed to indoor ULV space spray of Fludora Co-Max EW and K-Othrine EC.

**Additional file 14: Table S9.** Mortality of the wild insecticide-resistant *Aedes aegypti* and *Culex quinquefasciatus* Abidjan strain mosquitoes exposed to Fludora Co-Max EW and K-Othrine EC using indoor ULV space spray.

**Additional file 15: Table S10.** Knockdown rate at time intervals post-application in wild insecticide-resistant *Aedes aegypti* and *Culex quinquefasciatus* Abidjan strain mosquitoes exposed to indoor TF space spray of Fludora Co-Max EW and K-Othrine EC.

**Additional file 16: Table S11.** Mortality of the wild insecticide-resistant *Aedes aegypti* and *Culex quinquefasciatus* Abidjan strain mosquitoes exposed to Fludora Co-Max EW and K-Othrine EC using indoor TF space spray.

## Data Availability

Data generated or analysed during this study are included in this published article and its additional file. Additional data may be available from the corresponding author on reasonable request.

## Abbreviations

FFAST: Film Forming Aqueous Spray Technology
TF: Thermal fogging
ULV: Ultra-low volume
VMD: Volume medium diameter
