# Supplementary material for: Semi-field evaluation of the space spray efficacy of Fludora Co-Max EW against wild insecticide-resistant Aedes aegypti and Culex quinquefasciatus mosquito populations from Abidjan, Côte d’Ivoire
Source: Parasit Vectors. 2023 Feb 2;16:47. doi: 10.1186/s13071-022-05572-5 (PMC9893543; doi:10.1186/s13071-022-05572-5)
Supplement: Supplementary file 1 — Additional file 1: Table S1. Tentative dose calculation of the insecticide product formulation tested. [file 13071_2022_5572_MOESM1_ESM.docx]

| **Additional file 1: Table S1.** Tentative dose calculation of the insecticide product formulation tested. | | | | | |
| --- | --- | --- | --- | --- | --- |
| **Spraying method** | **Fludora Co-Max EW** | **Solvent** | **Replicate** | **Dilution Rate** | **Spraying volume** |
| Outdoor ULV cold fogging | Dose 1 | Water | 3 | 1:10 | 950 ml/ha |
| Outdoor thermal fogging | Dose 1 | Water | 3 | 1:50 | 4750 ml/ha |
| Indoor ULV cold fogging | Dose 1 | Water | 3 | 1:100 | 500 ml/1000 m^3^ |
| Indoor thermal fogging | Dose 1 | Water | 3 | 1:100 | 500 ml/1000 m^3^ |

Fludora Co-Max EC 78.8 needs to be mixed with water according to the table and calculation above. Some dosing information to achieve the targeted doses is provided below.

The label recommended active ingredient concentration for outdoor use is 7.5 g total a.i./ha.

The label recommended active ingredient concentration for indoor use is 0.394 g / 1000m^3^.

The a.i. content in Fludora Co-Max is 78.8 g total a.i./ liter.

1. ***Outdoor (Ultra Low Volume cold fogging)***

100ml Fludora Co-Max + 900ml water = (100x78.8)/1000 = 7.88 mg/ml; dose = 1:10

If 950ml of the solution were applied per hectare:

7.88 mg/ml x 950 ml = 7486 mg/ha (7.5 g/ha)

1. ***Outdoor (Thermal fogging)***

100ml Fludora Co-Max + 4900ml water = (100x78.8)/5000 = 1.576 mg/ml; dose = 1:50

If 4750ml of the solution were applied per hectare:

1.576 mg/ml x 4750 ml = 7486 mg/ha (7.5 g/ha)

1. ***Indoor (Ultra Low Volume cold fogging)***

10ml Fludora Co-Max + 990ml water = (10x78.8)/1000 = 0.788 mg/ml

If 500ml of the solution were applied per 1000m^3^:

0.788 mg/ml x 500 ml = 394 mg/1000m^3^ (0.394 g/1000m^3^)

1. ***Indoor (Thermal fogging)***

10ml Fludora Co-Max + 990ml water = (10x78.8)/1000 = 0.788 mg/ml

If 500ml of the solution were applied per 1000m^3^:

0.788 mg/ml x 500 ml = 394 mg/1000m^3^ (0.394 g/1000m^3^)
