# Supplementary figures and images for: Semi-field evaluation of the space spray efficacy of Fludora Co-Max EW against wild insecticide-resistant Aedes aegypti and Culex quinquefasciatus mosquito populations from Abidjan, Côte d’Ivoire
Source: Parasit Vectors. 2023 Feb 2;16:47. doi: 10.1186/s13071-022-05572-5 (PMC9893543; doi:10.1186/s13071-022-05572-5)

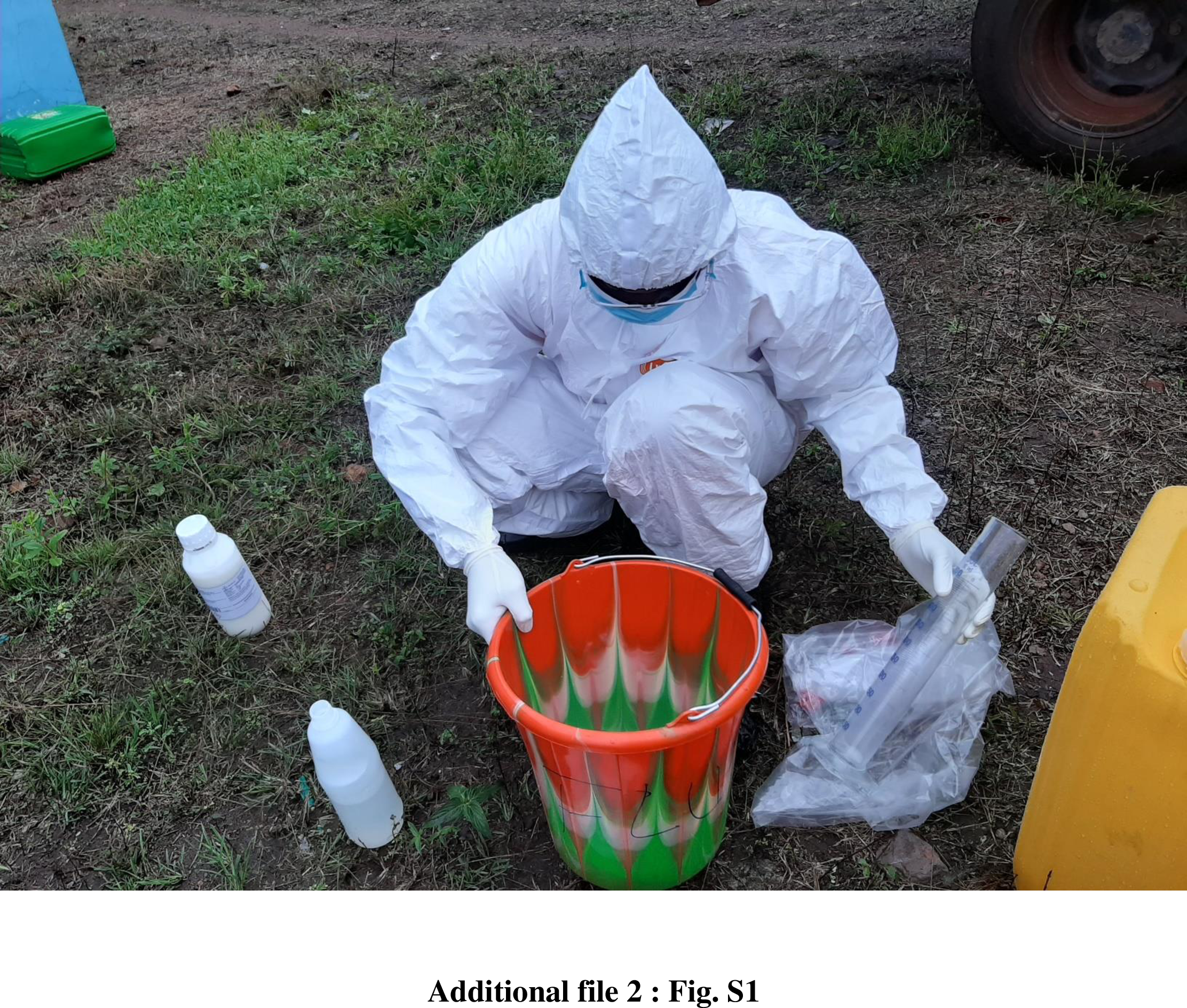

Supplement: Supplementary file 2 — Additional file 2: Figure S1. Dilution of Fludora Co-Max EW and K-Othrine performed in Agboville, Côte d’Ivoire. [file 13071_2022_5572_MOESM2_ESM.tif]

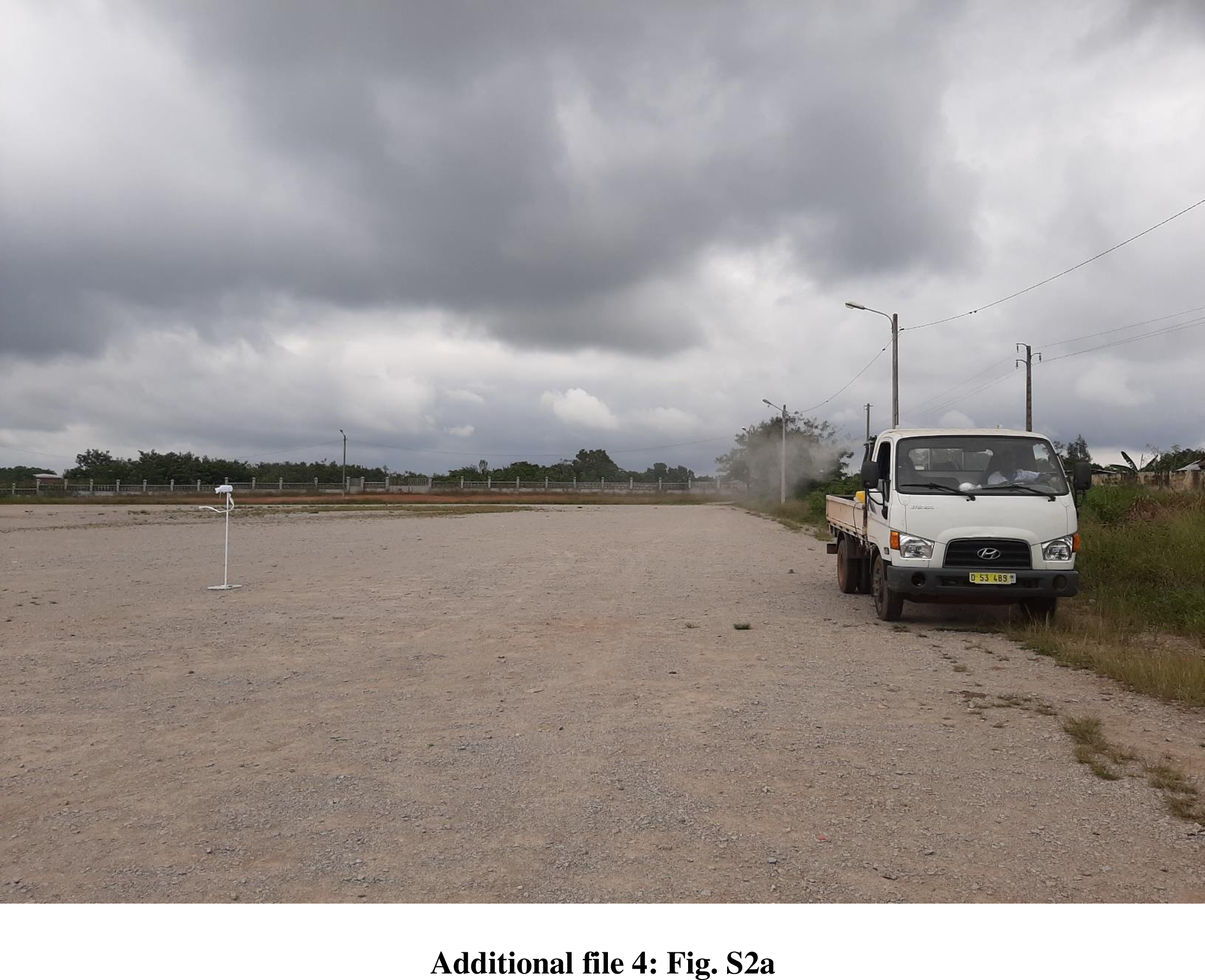

Supplement: Supplementary file 4 — Additional file 4: Figure S2. Different methods used for the semi-field evaluation of the efficacy of Fludora Co-Max EW and K-Othrine against Aedes aegypti and Culex quinquefasciatus Abidjan strain mosquitoes in Agboville, Côte d’Ivoire. a outdoor ULV, b outdoor TF, c indoor ULV, and d indoor TF. ULV, Ultra-low volume; TF, thermal fogging. [file 13071_2022_5572_MOESM4_ESM.zip › Additional file 4_Fig. S2a_29.10.2022.tif]

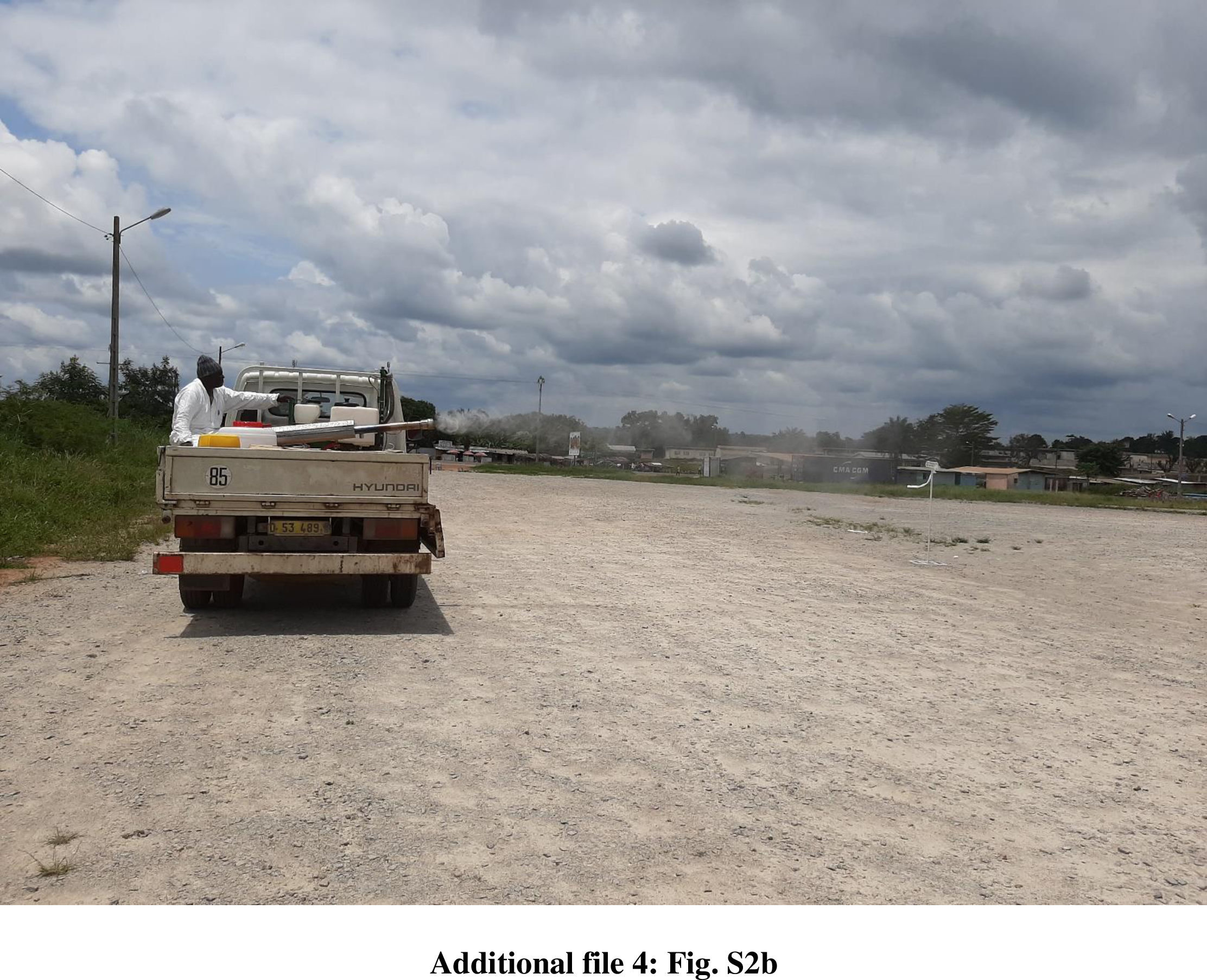

Supplement: Supplementary file 4 — Additional file 4: Figure S2. Different methods used for the semi-field evaluation of the efficacy of Fludora Co-Max EW and K-Othrine against Aedes aegypti and Culex quinquefasciatus Abidjan strain mosquitoes in Agboville, Côte d’Ivoire. a outdoor ULV, b outdoor TF, c indoor ULV, and d indoor TF. ULV, Ultra-low volume; TF, thermal fogging. [file 13071_2022_5572_MOESM4_ESM.zip › Additional file 4_Fig. S2b_29.10.2022.tif]

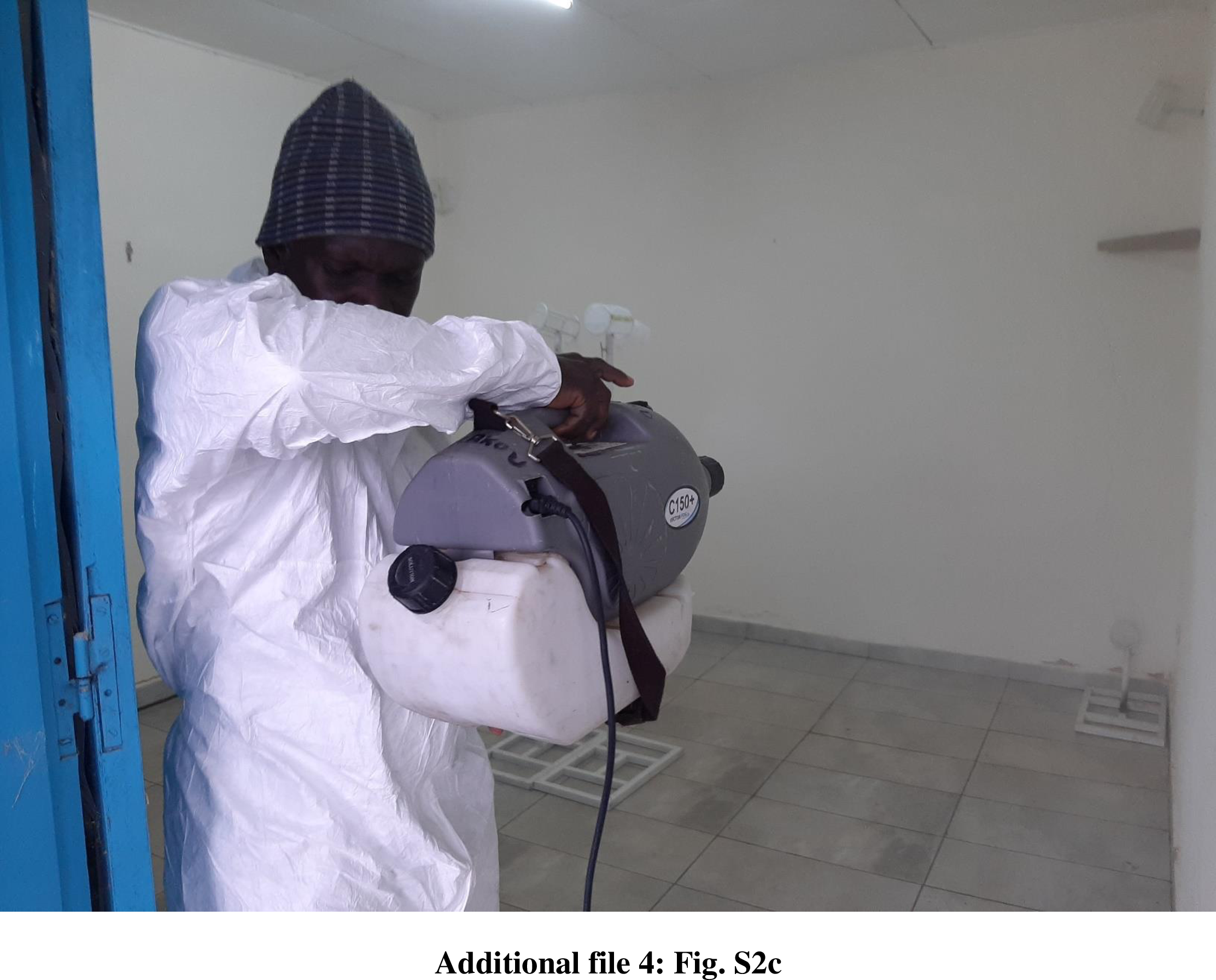

Supplement: Supplementary file 4 — Additional file 4: Figure S2. Different methods used for the semi-field evaluation of the efficacy of Fludora Co-Max EW and K-Othrine against Aedes aegypti and Culex quinquefasciatus Abidjan strain mosquitoes in Agboville, Côte d’Ivoire. a outdoor ULV, b outdoor TF, c indoor ULV, and d indoor TF. ULV, Ultra-low volume; TF, thermal fogging. [file 13071_2022_5572_MOESM4_ESM.zip › Additional file 4_Fig. S2c_29.10.2022.tif]

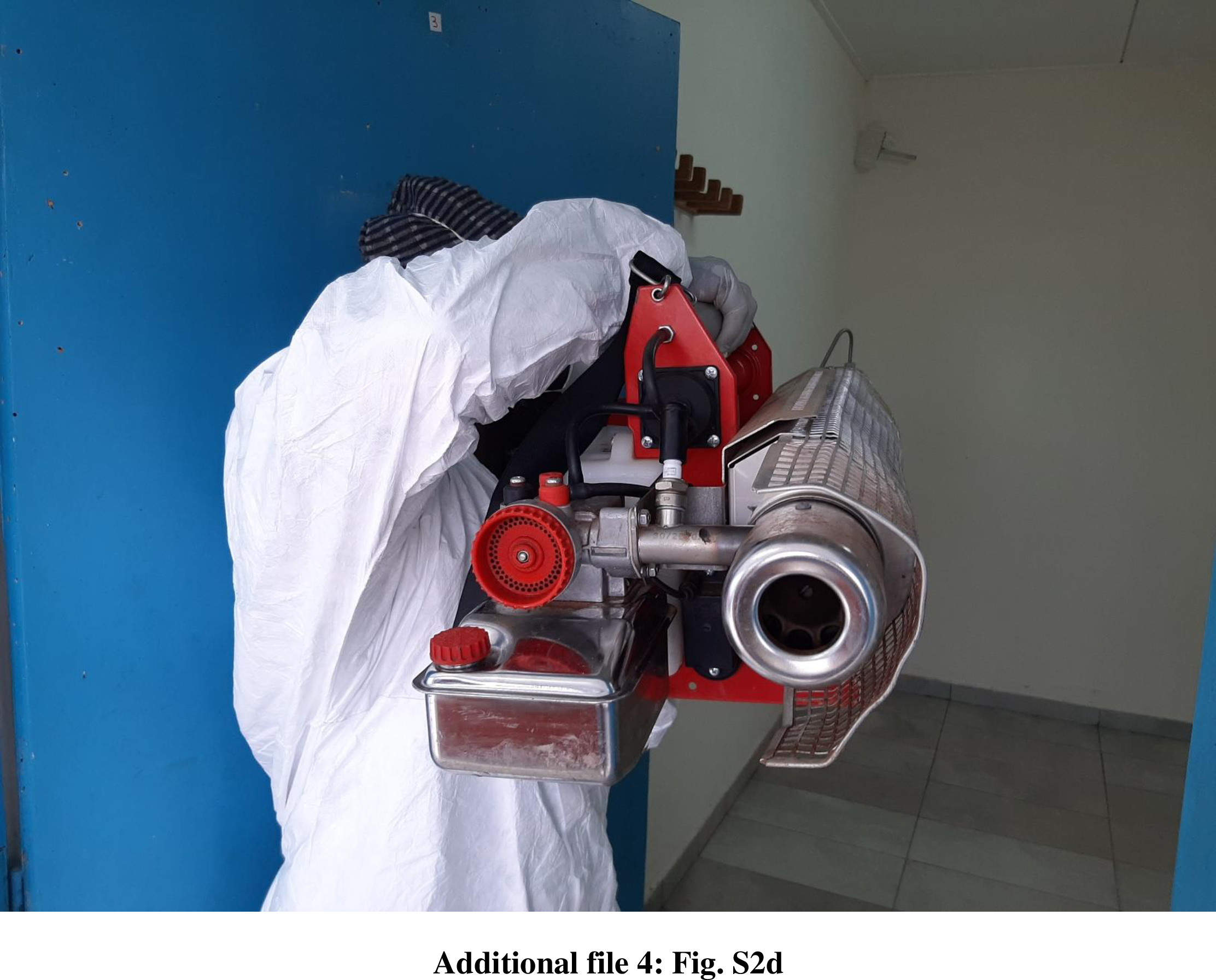

Supplement: Supplementary file 4 — Additional file 4: Figure S2. Different methods used for the semi-field evaluation of the efficacy of Fludora Co-Max EW and K-Othrine against Aedes aegypti and Culex quinquefasciatus Abidjan strain mosquitoes in Agboville, Côte d’Ivoire. a outdoor ULV, b outdoor TF, c indoor ULV, and d indoor TF. ULV, Ultra-low volume; TF, thermal fogging. [file 13071_2022_5572_MOESM4_ESM.zip › Additional file 4_Fig. S2d_29.10.2022.tif]

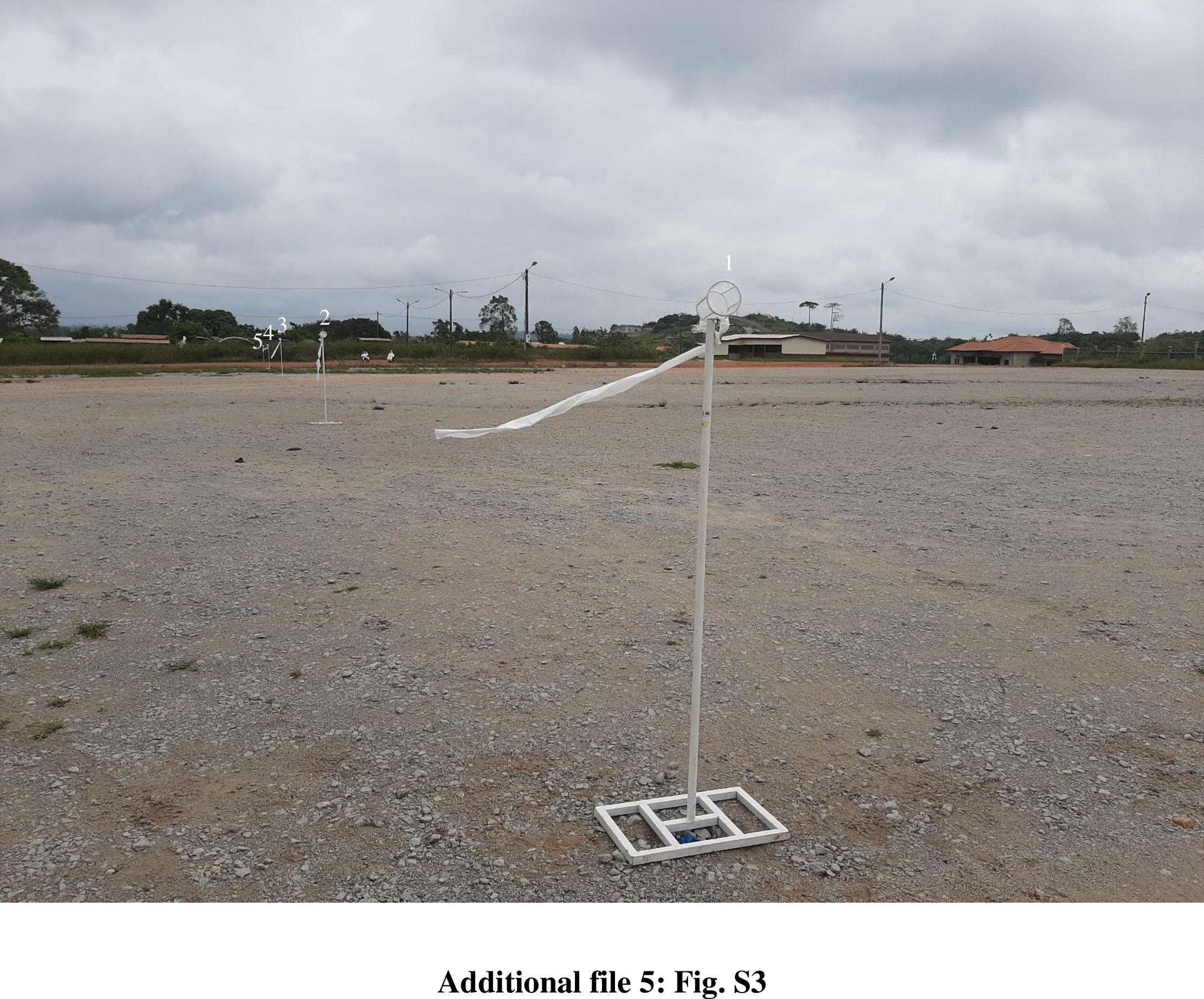

Supplement: Supplementary file 5 — Additional file 5: Figure S3. Outdoor trial semi-field station with 1.5-m tall poles placed at five different distance checkpoints. 1, 10 m; 2, 25 m; 3, 50 m; 4, 75 m; 5; 100 m. [file 13071_2022_5572_MOESM5_ESM.tif]

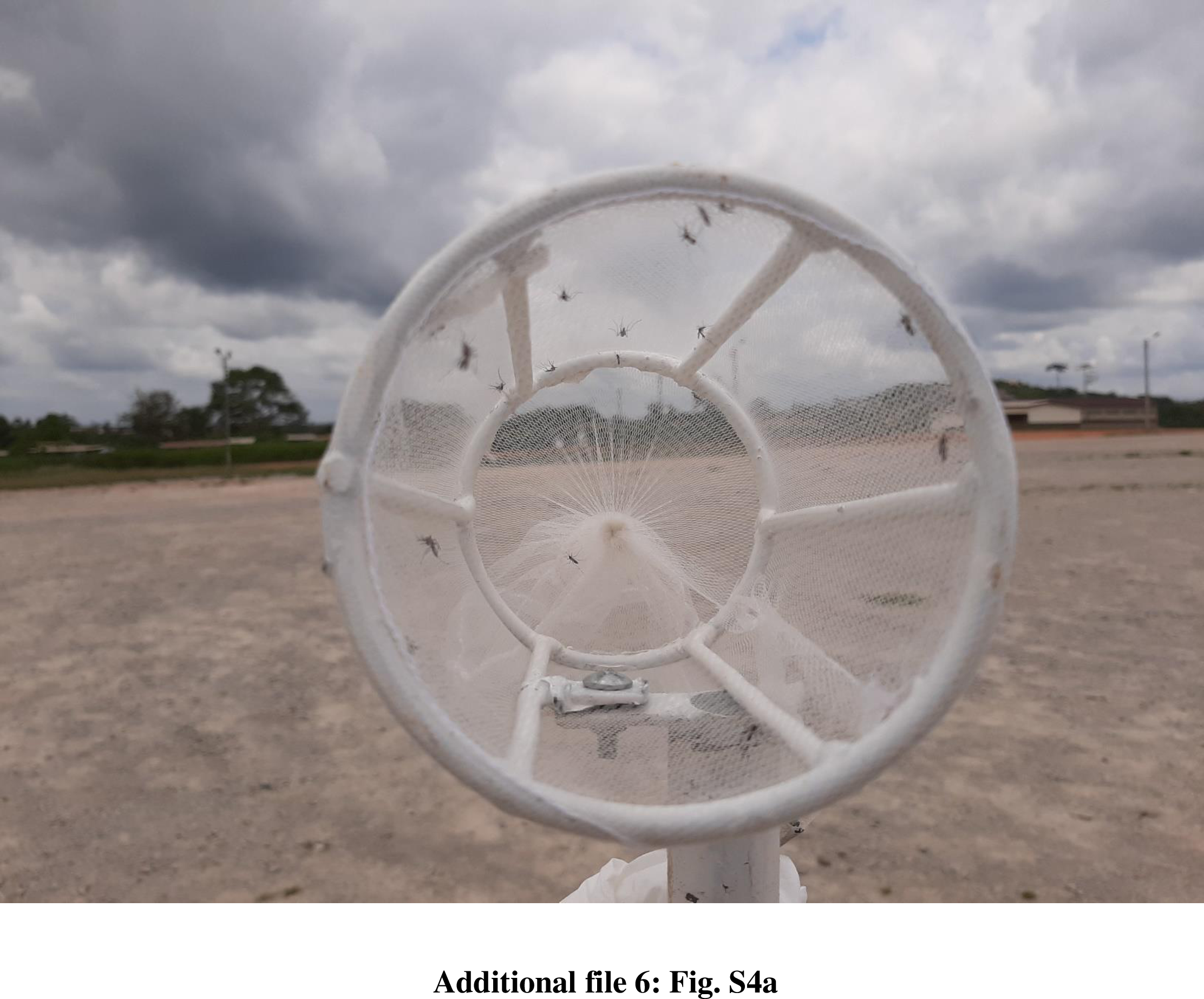

Supplement: Supplementary file 6 — Additional file 6: Figure S4. Cylindrical cages constructed of fine mesh fabric (nylon) with wire frame support (diameter 10 cm × height 15 cm × tapping cover 10 cm) and containing adult mosquitoes for outdoor trial. a Front view, b profile view. [file 13071_2022_5572_MOESM6_ESM.zip › Additional file 6_Fig S4a_29.10.2022.tif]

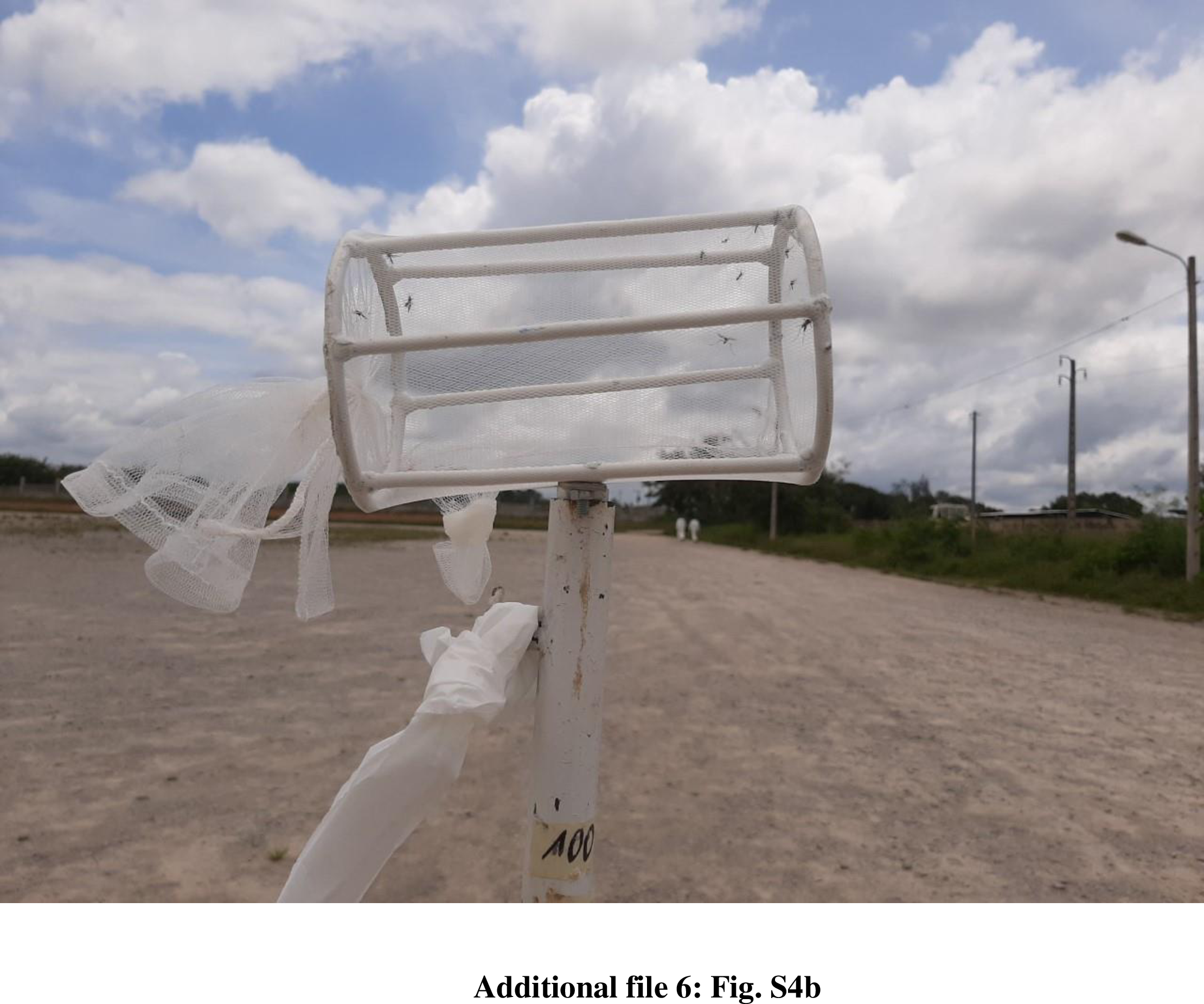

Supplement: Supplementary file 6 — Additional file 6: Figure S4. Cylindrical cages constructed of fine mesh fabric (nylon) with wire frame support (diameter 10 cm × height 15 cm × tapping cover 10 cm) and containing adult mosquitoes for outdoor trial. a Front view, b profile view. [file 13071_2022_5572_MOESM6_ESM.zip › Additional file 6_Fig S4b_29.10.2022.tif]

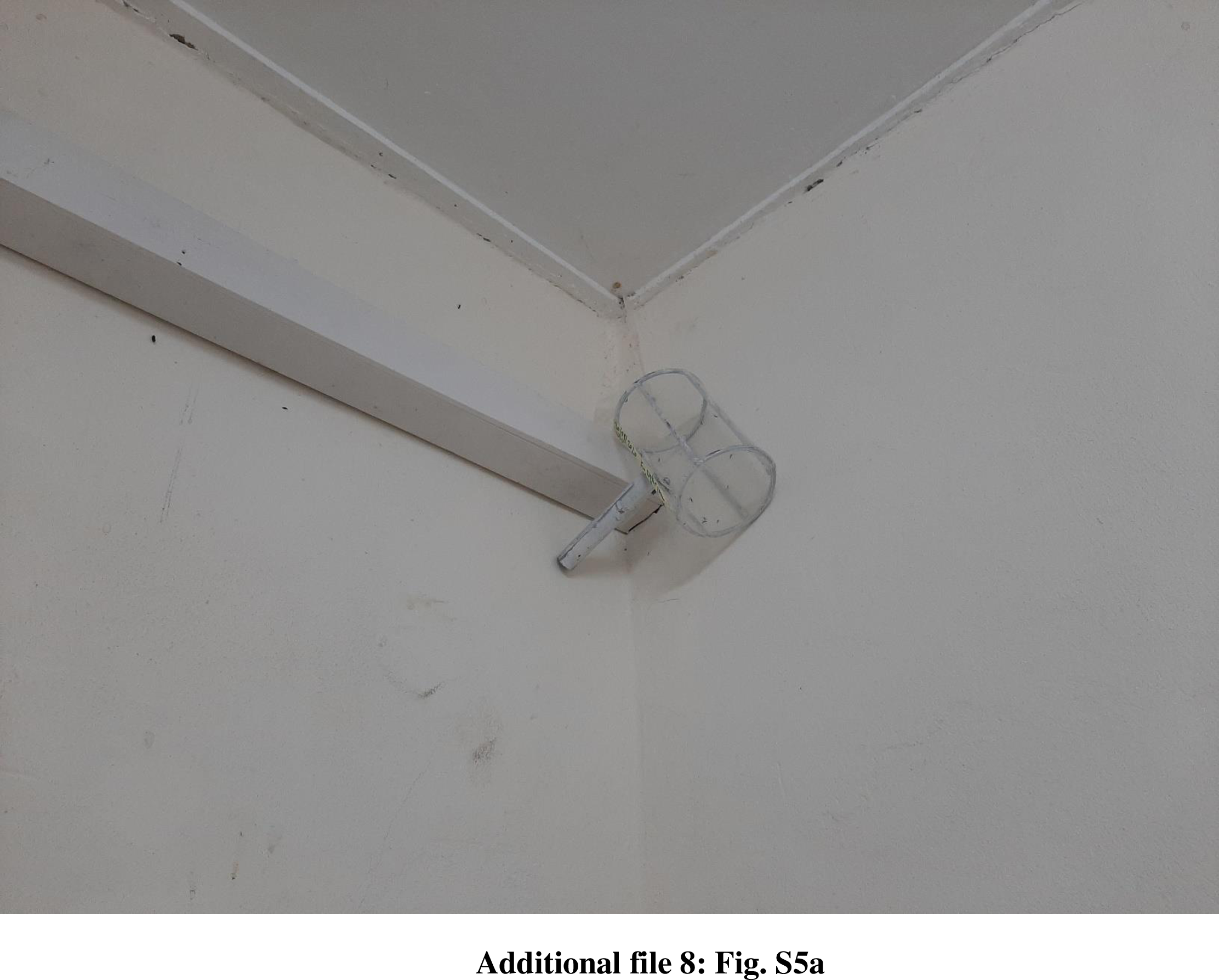

Supplement: Supplementary file 8 — Additional file 8: Figure S5. Indoor trial semi-field station with mosquito cages installed at different level checkpoints in a house. a Ceiling, b mid-height, c floor. [file 13071_2022_5572_MOESM8_ESM.zip › Additional file 8_Fig S5a_29.10.2022.tif]

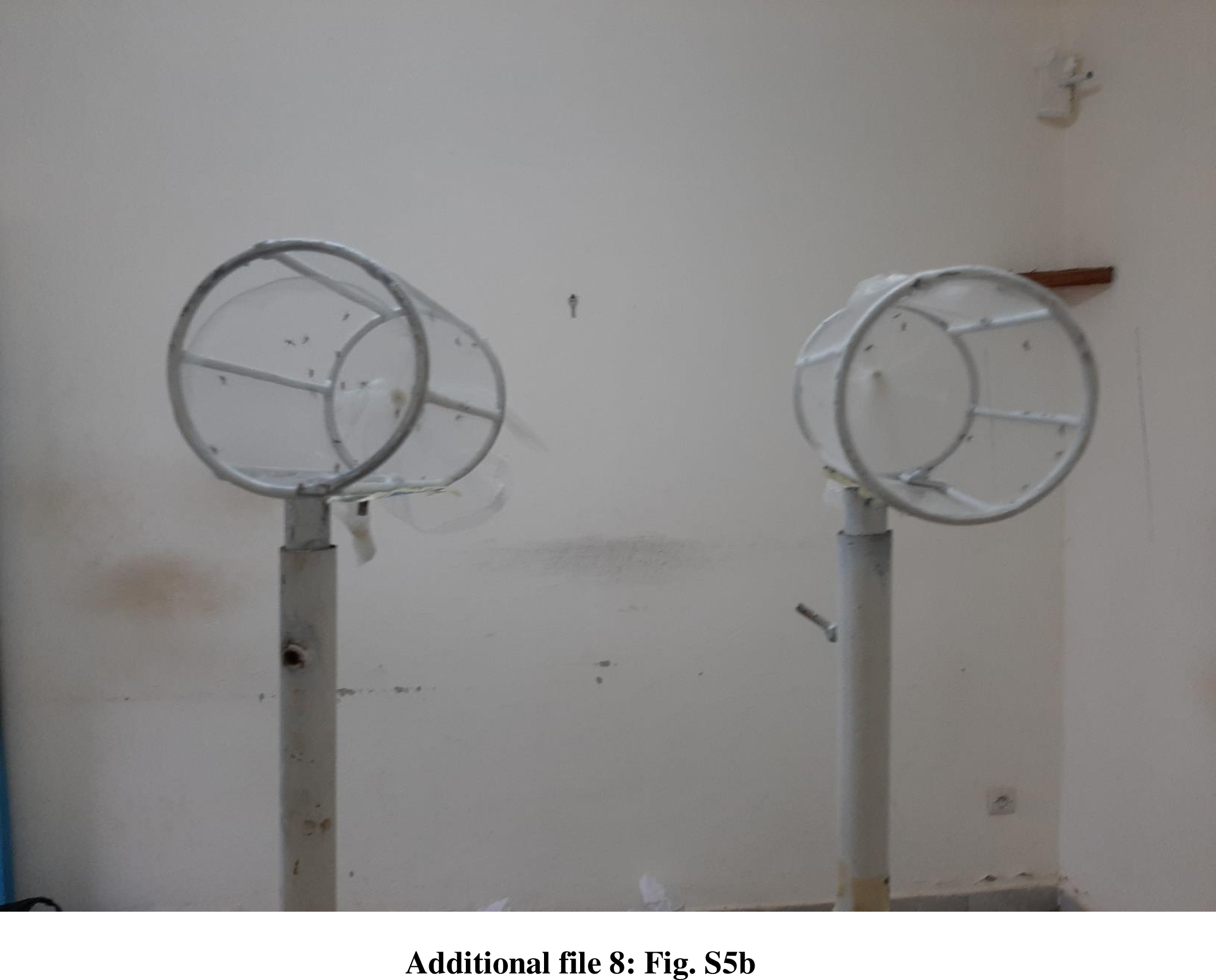

Supplement: Supplementary file 8 — Additional file 8: Figure S5. Indoor trial semi-field station with mosquito cages installed at different level checkpoints in a house. a Ceiling, b mid-height, c floor. [file 13071_2022_5572_MOESM8_ESM.zip › Additional file 8_Fig S5b_29.10.2022.tif]

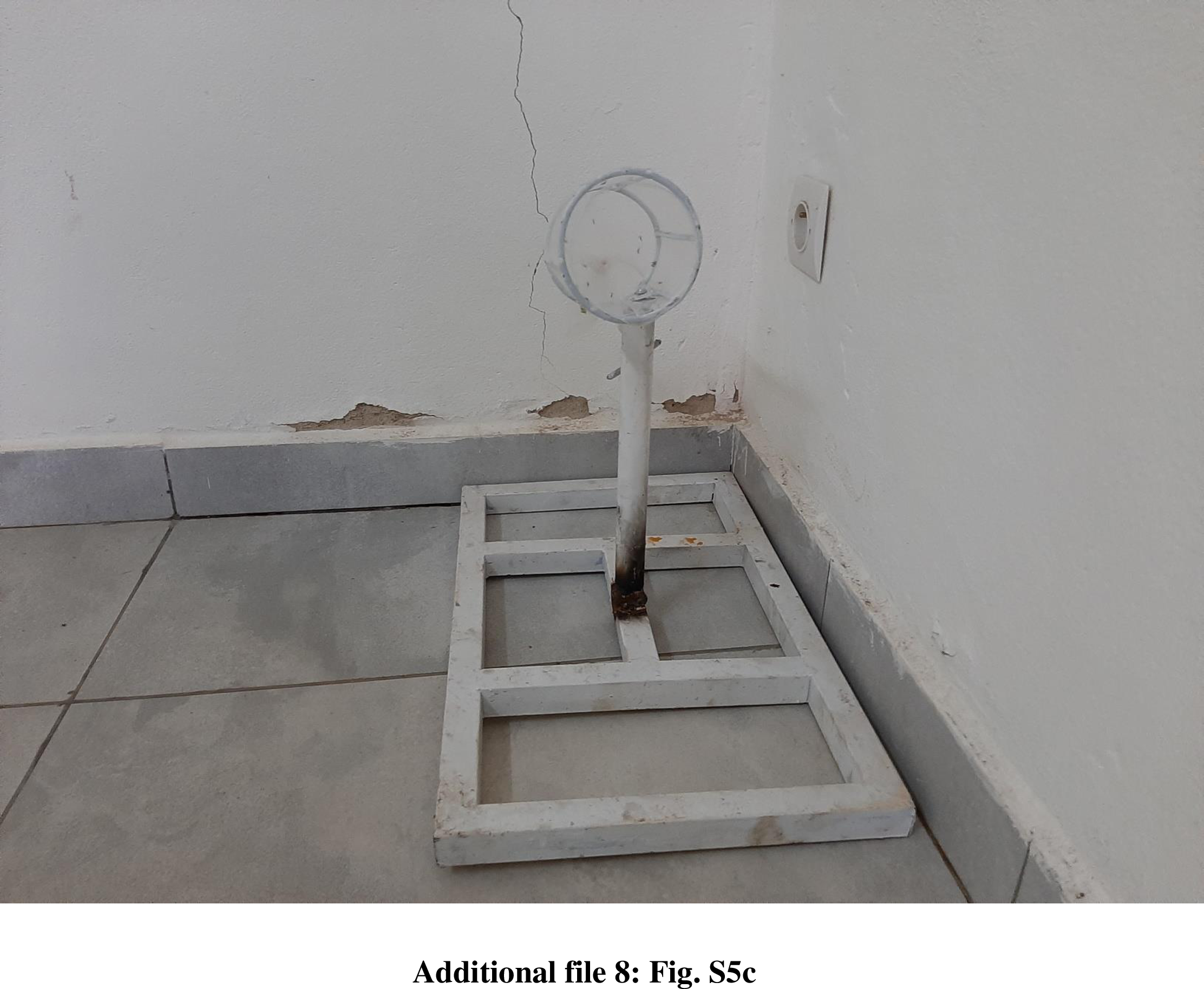

Supplement: Supplementary file 8 — Additional file 8: Figure S5. Indoor trial semi-field station with mosquito cages installed at different level checkpoints in a house. a Ceiling, b mid-height, c floor. [file 13071_2022_5572_MOESM8_ESM.zip › Additional file 8_Fig S5c_29.10.2022.tif]
