# Supplementary material for: Semi-field evaluation of the space spray efficacy of Fludora Co-Max EW against wild insecticide-resistant Aedes aegypti and Culex quinquefasciatus mosquito populations from Abidjan, Côte d’Ivoire
Source: Parasit Vectors. 2023 Feb 2;16:47. doi: 10.1186/s13071-022-05572-5 (PMC9893543; doi:10.1186/s13071-022-05572-5)
Supplement: Supplementary file 3 — Additional file 3: Table S2. Meteorological data recorded in the field during the small-scale trials for testing Fludora Co-Max EW and K-Othrine EC against Aedes aegypti and Culex quinquefasciatus in Agboville, Côte d’Ivoire. [file 13071_2022_5572_MOESM3_ESM.docx]

| **Additional file 3: Table S2.** Meteorological data recorded during the small-scale field trials for testing Fludora Co-Max EW and K-Othrine EC against *Aedes aegypti* and *Culex quinquefasciatus* in Agboville, Côte d’Ivoire | | | | | | | | | | |
| --- | --- | --- | --- | --- | --- | --- | --- | --- | --- | --- |
| **Method** | **Place** | **Product** | **Replicate** | **Date** | **Time** | | **T”C** | | **%RH** | |
|  |  |  |  |  | ***Start*** | ***End*** | ***Start*** | ***End*** | ***Start*** | ***End*** |
| ***Aedes aegypti*** | | | | | | | | | | |
| ULV | Outdoors | Control (water) | 1 | 20/09/2020 | 08:13 | 08:28 | 26.4 | 27.0 | 70.5 | 72.3 |
| ULV | Outdoors | Fludora Co-Max EW | 1 | 20/09/2020 | 09:26 | 09:41 | 27.9 | 28.4 | 67.4 | 69.1 |
| ULV | Outdoors | K-Othrine EC | 1 | 20/09/2020 | 10:28 | 10:43 | 29.9 | 30.2 | 68.0 | 66.8 |
| ULV | Outdoors | Control (water) | 2 | 21/09/2020 | 09:17 | 09:33 | 28.3 | 29.1 | 70.7 | 70.2 |
| ULV | Outdoors | Fludora Co-Max EW | 2 | 21/09/2020 | 10:19 | 10:34 | 29.5 | 29.1 | 69.0 | 68.0 |
| ULV | Outdoors | K-Othrine EC | 2 | 21/09/2020 | 11:25 | 11:41 | 28.1 | 28.8 | 66.5 | 65,7 |
| ULV | Outdoors | Control (water) | 3 | 30/09/2020 | 16:08 | 16:23 | 30.8 | 30.9 | 68.2 | 69.1 |
| ULV | Outdoors | Fludora Co-Max EW | 3 | 30/09/2020 | 17:13 | 17:28 | 27.5 | 27.4 | 72.9 | 73.7 |
| ULV | Outdoors | K-Othrine EC | 3 | 30/09/2020 | 18:14 | 19:20 | 27.2 | 27.2 | 76.7 | 78.3 |
|  |  |  |  |  |  |  |  |  |  |  |
| TF | Outdoors | Control (water) | 1 | 20/09/2020 | 16:03 | 16:18 | 30.3 | 29.8 | 73.8 | 72.5 |
| TF | Outdoors | Fludora Co-Max EW | 1 | 20/09/2020 | 16:55 | 17:10 | 28.1 | 28.2 | 72.2 | 73.1 |
| TF | Outdoors | K-Othrine EC | 1 | 20/09/2020 | 18:13 | 18:28 | 26.1 | 25.2 | 76.2 | 77.9 |
| TF | Outdoors | Control (water) | 2 | 21/09/2020 | 06:06 | 06:21 | 24.3 | 25.8 | 77.7 | 78.2 |
| TF | Outdoors | Fludora Co-Max EW | 2 | 21/09/2020 | 07:11 | 07:26 | 27.5 | 28.1 | 73.0 | 70.1 |
| TF | Outdoors | K-Othrine EC | 2 | 21/09/2020 | 08:17 | 08:33 | 28.1 | 28.8 | 74.5 | 73,7 |
| TF | Outdoors | Control (water) | 3 | 30/09/2020 | 06:16 | 06:32 | 27.3 | 27.1 | 73.2 | 73.9 |
| TF | Outdoors | Fludora Co-Max EW | 3 | 30/09/2020 | 07:20 | 07:35 | 28.4 | 28.5 | 65.4 | 60.6 |
| TF | Outdoors | K-Othrine EC | 3 | 30/09/2020 | 08:29 | 08:45 | 28.6 | 28.6 | 58.3 | 56.2 |
|  |  |  |  |  |  |  |  |  |  |  |
| ULV | Indoors | Control (water) | 1 | 20/09/2020 | 06:35 | 06:50 | 26.8 | 27.1 | 72.2 | 70.4 |
| ULV | Indoors | Fludora Co-Max EW | 1 | 20/09/2020 | 07:37 | 07:52 | 27.5 | 27.6 | 71.9 | 72.4 |
| ULV | Indoors | K-Othrine EC | 1 | 20/09/2020 | 08:51 | 09:06 | 28.4 | 28.3 | 70.8 | 68.9 |
| ULV | Indoors | Control (water) | 2 | 21/09/2020 | 09:30 | 09:45 | 31.3 | 31.5 | 64.2 | 55.9 |
| ULV | Indoors | Fludora Co-Max EW | 2 | 21/09/2020 | 10:34 | 10:49 | 31.8 | 32.0 | 62.6 | 55.3 |
| ULV | Indoors | K-Othrine EC | 2 | 21/09/2020 | 11:35 | 11:51 | 31.9 | 31.7 | 54.1 | 53.0 |
| ULV | Indoors | Control (water) | 3 | 30/09/2020 | 09:01 | 09:17 | 30.1 | 29.3 | 68.6 | 69.2 |
| ULV | Indoors | Fludora Co-Max EW | 3 | 30/09/2020 | 10:08 | 10:24 | 30.2 | 30.1 | 65.3 | 64.8 |
| ULV | Indoors | K-Othrine EC | 3 | 30/09/2020 | 11:26 | 11:41 | 30.1 | 30.3 | 62.1 | 60.3 |
|  |  |  |  |  |  |  |  |  |  |  |
| TF | Indoors | Control (water) | 1 | 20/09/2020 | 09:08 | 10:23 | 29.9 | 30.1 | 61.1 | 57.2 |
| TF | Indoors | Fludora Co-Max EW | 1 | 20/09/2020 | 10:12 | 08:39 | 30.3 | 30.2 | 56.0 | 53.8 |
| TF | Indoors | K-Othrine EC | 1 | 20/09/2020 | 11:14 | 12:46 | 29.9 | 30.1 | 59.1 | 55.9 |
| TF | Indoors | Control (water) | 2 | 21/09/2020 | 07:02 | 07:18 | 26.3 | 26.8 | 74.2 | 73.9 |
| TF | Indoors | Fludora Co-Max EW | 2 | 21/09/2020 | 08:21 | 08:36 | 26.6 | 28.3 | 72.6 | 73.3 |
| TF | Indoors | K-Othrine EC | 2 | 21/09/2020 | 09:25 | 09:40 | 28.2 | 29.1 | 72.1 | 70.3 |
| TF | Indoors | Control (water) | 3 | 30/09/2020 | 09:40 | 09:55 | 30.7 | 30.7 | 55.4 | 57.9 |
| TF | Indoors | Fludora Co-Max EW | 3 | 30/09/2020 | 10:40 | 10:56 | 30.9 | 30.6 | 53.8 | 58.9 |
| TF | Indoors | K-Othrine EC | 3 | 30/09/2020 | 11:41 | 11:56 | 31.8 | 32.1 | 56.3 | 55.1 |

*%RH: Relative humidity (%), T°C: Temperature (°C), TF: Thermal fogging, ULV: Ultra-low volume*

| **Method** | **Place** | **Product** | **Replicate** | **Date** | **Time** | | **T°C** | | **%RH** | |
| --- | --- | --- | --- | --- | --- | --- | --- | --- | --- | --- |
|  |  |  |  |  | ***Start*** | ***End*** | ***Start*** | ***End*** | ***Start*** | ***End*** |
| ***Culex quinquefasciatus*** | | | | | | | | | | |
| ULV | Outdoors | Fludora Co-Max EW | 1 | 05/12/2020 | 17:08 | 17:23 | 30.3 | 29.9 | 70.0 | 68.0 |
| ULV | Outdoors | K-Othrine EC | 1 | 05/12/2020 | 18:13 | 18:29 | 28.3 | 28.1 | 73.2 | 78.7 |
| ULV | Outdoors | Control (water) | 2 | 13/12/2020 | 08:06 | 08:21 | 28.6 | 29.2 | 73,7 | 70,3 |
| ULV | Outdoors | Fludora Co-Max EW | 2 | 13/12/2020 | 09:45 | 10:00 | 30,3 | 31.0 | 53,9 | 52.5 |
| ULV | Outdoors | K-Othrine EC | 2 | 13/12/2020 | 10:33 | 17:48 | 31,6 | 32.1 | 51.1 | 50.7 |
| ULV | Outdoors | Control (water) | 3 | 14/12/2020 | 07:16 | 07:31 | 29.4 | 30.1 | 74.7 | 76.2 |
| ULV | Outdoors | Fludora Co-Max EW | 3 | 14/12/2020 | 08:17 | 08:33 | 30.8 | 31.0 | 73.1 | 71.8 |
| ULV | Outdoors | K-Othrine EC | 3 | 14/12/2020 | 09:20 | 09:35 | 31.4 | 31.5 | 60.0 | 58.6 |
|  |  |  |  |  |  |  |  |  |  |  |
| TF | Outdoors | Control (water) | 1 | 06/12/2020 | 08:06 | 08:21 | 27.1 | 26.9 | 73,7 | 70,3 |
| TF | Outdoors | Fludora Co-Max EW | 1 | 06/12/2020 | 09:12 | 09:27 | 26,3 | 28.3 | 66,9 | 62.5 |
| TF | Outdoors | K-Othrine EC | 1 | 06/12/2020 | 10:33 | 17:48 | 29,1 | 29.2 | 61.1 | 60.7 |
| TF | Outdoors | Control (water) | 2 | 13/12/2020 | 16:02 | 16:18 | 32.6 | 32.2 | 57,7 | 64,3 |
| TF | Outdoors | Fludora Co-Max EW | 2 | 13/12/2020 | 17:10 | 17:26 | 30,3 | 29.0 | 73,9 | 73.5 |
| TF | Outdoors | K-Othrine EC | 2 | 13/12/2020 | 18:13 | 18:28 | 28,6 | 28.1 | 76.3 | 78.7 |
| TF | Outdoors | Control (water) | 3 | 14/12/2020 | 16:21 | 16:36 | 32.1 | 31.2 | 71.7 | 67.9 |
| TF | Outdoors | Fludora Co-Max EW | 3 | 14/12/2020 | 17:55 | 17:11 | 30.8 | 29.1 | 69.1 | 61.8 |
| TF | Outdoors | K-Othrine EC | 3 | 14/12/2020 | 18:57 | 18:12 | 27.8 | 27.6 | 57.0 | 55.6 |
|  |  |  |  |  |  |  |  |  |  |  |
| ULV | Indoors | Control (water) | 1 | 06/12/2020 | 07:01 | 08:16 | 26.8 | 26.9 | 75.4 | 74.5 |
| ULV | Indoors | Fludora Co-Max EW | 1 | 06/12/2020 | 08:06 | 08:21 | 27.1 | 26.8 | 74.1 | 72.2 |
| ULV | Indoors | K-Othrine EC | 1 | 06/12/2020 | 09:22 | 09:37 | 30.6 | 30.7 | 68.9 | 65.4 |
| ULV | Indoors | Control (water) | 2 | 13/12/2020 | 07:15 | 07:30 | 26.3 | 26.2 | 79,7 | 76,3 |
| ULV | Indoors | Fludora Co-Max EW | 2 | 13/12/2020 | 08:22 | 08:37 | 27,3 | 28.0 | 73,2 | 71.0 |
| ULV | Indoors | K-Othrine EC | 2 | 13/12/2020 | 09:25 | 09:40 | 30,6 | 31.0 | 56.1 | 53.7 |
| ULV | Indoors | Control (water) | 3 | 14/12/2020 | 07:05 | 07:21 | 25.6 | 25.9 | 78.7 | 76.3 |
| ULV | Indoors | Fludora Co-Max EW | 3 | 14/12/2020 | 08:12 | 08:27 | 28,1 | 27.8 | 72.5 | 70.1 |
| ULV | Indoors | K-Othrine EC | 3 | 14/12/2020 | 09:12 | 09:27 | 30.9 | 31.2 | 66,7 | 61.2 |
|  |  |  |  |  |  |  |  |  |  |  |
| TF | Indoors | Control (water) | 1 | 06/12/2020 | 10:23 | 10:38 | 30.8 | 31.0 | 62.4 | 58.5 |
| TF | Indoors | Fludora Co-Max EW | 1 | 06/12/2020 | 11:26 | 11:41 | 31.8 | 31.9 | 54.1 | 52.2 |
| TF | Indoors | K-Othrine EC | 1 | 06/12/2020 | 12:32 | 09:47 | 32.6 | 32.7 | 51.9 | 50.4 |
| TF | Indoors | Control (water) | 2 | 13/12/2020 | 10:27 | 10:42 | 31.3 | 31.0 | 53,7 | 55,3 |
| TF | Indoors | Fludora Co-Max EW | 2 | 13/12/2020 | 11:28 | 11:44 | 31,3 | 32.0 | 54,2 | 52.6 |
| TF | Indoors | K-Othrine EC | 2 | 13/12/2020 | 12:30 | 12:45 | 32,1 | 32.2 | 51.5 | 50.1 |
| TF | Indoors | Control (water) | 3 | 14/12/2020 | 10:14 | 10:39 | 31.3 | 31.5 | 57.7 | 52.2 |
| TF | Indoors | Fludora Co-Max EW | 3 | 14/12/2020 | 11:16 | 11:23 | 32.3 | 32.0 | 55.1 | 53.8 |
| TF | Indoors | K-Othrine EC | 3 | 14/12/2020 | 12:21 | 12:36 | 33.3 | 33.4 | 52.0 | 51.6 |

*%RH: Relative humidity (%), T°C: Temperature (°C), TF: Thermal fogging, ULV: Ultra-low volume*
