# Supplementary material for: Semi-field evaluation of the space spray efficacy of Fludora Co-Max EW against wild insecticide-resistant Aedes aegypti and Culex quinquefasciatus mosquito populations from Abidjan, Côte d’Ivoire
Source: Parasit Vectors. 2023 Feb 2;16:47. doi: 10.1186/s13071-022-05572-5 (PMC9893543; doi:10.1186/s13071-022-05572-5)
Supplement: Supplementary file 7 — Additional file 7: Table S3. Parameters of calibration of the sprayers for outdoor and indoor application against Aedes aegypti and Culex quinquefasciatus in Agboville, Côte d’Ivoire. [file 13071_2022_5572_MOESM7_ESM.docx]

| **Additional file 7: Table S3.** Parameters of calibration of the sprayers for outdoor and indoor application against *Aedes aegypti* and *Culex quinquefasciatus* in Agboville, Côte d’Ivoire | | | |
| --- | --- | --- | --- |
| **Sprayer** | **Parameters** | **Fludora Co-Max EW** | **K-Othrine EC** |
| Micronair AU9000 (Outdoor ULV) | Track spacing (m) | 50 | 50 |
|  | Vehicle speed (Km/h) | 12 | 12 |
|  | Application rate (l/ha) | 0.95 | 1 |
|  | Coverage (ha/min) | 1 | 1 |
|  | Flow from sprayer (l/min) | 0.95 | 1 |
|  | Number of sprayhead | 2 | 2 |
|  | Output from each sprayhead (l/min) | 0.475 | 0.5 |
|  | Switch position or Nozzle | 6 | 6 |
|  | Required flow rate (l/min) | 0.5 | 0.5 |
|  | Volume from right-hand sprayhead (l) | 0.53 | 0.49 |
|  | Volume from left-hand sprayhead (l) | 0.45 | 0.47 |
|  | Total volume (l) | 0.98 | 0.96 |
|  | Time to collect measured volume (min) | 1 | 1 |
|  | Flow rate (l/min) | 0.98 | 0.96 |
|  |  |  |  |
| Swingfog SN 101 Pump (Outdoor TF) | Track spacing (m) | 25 | 25 |
|  | Vehicle speed (km/h) | 10 | 10 |
|  | Application rate (l/ha) | 4.75 | 5 |
|  | Coverage (ha/min) | 0.42 | 0.42 |
|  | Flow from sprayer (l/min) | 1.98 | 2.08 |
|  | Number of sprayhead | 1 | 1 |
|  | Switch position or Nozzle | 3 | 4.5 |
|  | Required flow rate (l/min) | 1.98 | 2.1 |
|  | Volume from sprayhead (l) | 1.91 | 1.95 |
|  | Time to collect measured volume (min) | 1 | 1 |
|  | Flow rate (l/min) | 1.91 | 1.95 |
|  |  |  |  |
| Vectorfog C150+ (Indoor ULV) | Flow rate (l/min) | 0.83 | 0.81 |
|  | House volume (m^3^) | 126 | 126 |
|  | Application rate (ml/m^3^) | 0.5 | 0.5 |
|  | Volume (ml) | 63 | 63 |
|  | Time (min or sec) | 0.076 min or 4 sec | 0.076 min or 4 sec |
|  |  |  |  |
| Swingfog SN 50 (Indoor TF) | Flow rate (l/min) | 0.17 | 0.16 |
|  | House volume (m^3^) | 126 | 126 |
|  | Application rate (l/ m^3^) | 0.5 | 0.5 |
|  | Volume (ml) | 63 | 63 |
|  | Time (min or sec) | 0.38 min or 23 sec | 0.38 min or 23 sec |
| Formula:  $Coverage (ha/min)=\frac{Track spacing \left( m \right) x Vehicle speed (km/h)}{600}$  $Flow from sprayer (l/min) = Coverage (ha/min) x Application rate (l/ha)$  $Time (min)=\frac{Total volume \left( l \right)}{Flow rate (l/min)}$  *Ha: hectare, L: litre, m: meter, min: minute, km: kilometre, sec: second, ULV: ultra-low volume, TF: thermal fogging* | | | |
