# Supplementary material for: Semi-field evaluation of the space spray efficacy of Fludora Co-Max EW against wild insecticide-resistant Aedes aegypti and Culex quinquefasciatus mosquito populations from Abidjan, Côte d’Ivoire
Source: Parasit Vectors. 2023 Feb 2;16:47. doi: 10.1186/s13071-022-05572-5 (PMC9893543; doi:10.1186/s13071-022-05572-5)
Supplement: Supplementary file 9 — Additional file 9: Table S4. Knockdown rate at time intervals post-application in the wild insecticide-resistant Aedes aegypti and Culex quinquefasciatus Abidjan strain mosquitoes exposed to outdoor ULV space spray of Fludora Co-Max EW and K-Othrine EC. ULV, ultra-low volume. [file 13071_2022_5572_MOESM9_ESM.docx]

| **Additional file 9: Table S4** Knockdown rate (%) at time intervals post-application in the wild insecticide-resistant *Aedes aegypti* and *Culex quinquefasciatus* Abidjan strains exposed to outdoor ULV space spray of Fludora Co-Max EW and K-Othrine EC | | | | | | | | | | | | | | | |
| --- | --- | --- | --- | --- | --- | --- | --- | --- | --- | --- | --- | --- | --- | --- | --- |
| **Treatment arm** | **Checkpoint** | ***Aedes aegypti*** | | | | | | | ***Culex quinquefasciatus*** | | | | | | |
|  |  | **0 min** | **10 min** | **20 min** | **30 min** | **40 min** | **50 min** | **60 min** | **0 min** | **10 min** | **20 min** | **30 min** | **40 min** | **50 min** | **60 min** |
| **Fludora Co-Max EW** | 10 m | 100 | 100 | 100 | 100 | 100 | 100 | 100 | 100 | 100 | 100 | 100 | 100 | 100 | 100 |
|  | 25 m | 96.7 | 96.7 | 98.3 | 100 | 100 | 100 | 100 | 100 | 100 | 100 | 100 | 100 | 100 | 100 |
|  | 50 m | 83.3 | 85.0 | 96.7 | 98.3 | 96.7 | 100 | 98.0 | 91.7 | 88.3 | 96.7 | 98.3 | 100 | 100 | 100 |
|  | 75 m | 88.3 | 91.7 | 91.7 | 95.0 | 96.7 | 98.3 | 100 | 63.3 | 63.3 | 80.0 | 91.7 | 96.7 | 96.7 | 100 |
|  | 100 m | 85.0 | 85.0 | 88.3 | 86.7 | 90.0 | 95.0 | 97.7 | 53.3 | 53.3 | 66.7 | 85.0 | 91.7 | 93.3 | 95.0 |
|  | **Total** | **90.7** | **91.7** | **95.0** | **96.0** | **96.7** | **98.7** | **99.1** | **81.7** | **81.0** | **88.7** | **95.0** | **97.7** | **98.0** | **99.0** |
|  |  |  |  |  |  |  |  |  |  |  |  |  |  |  |  |
| **K-Othrine EC** | 10 m | 83.3 | 83.3 | 83.3 | 88.3 | 86.7 | 91.7 | 91.7 | 96.7 | 96.7 | 96.7 | 98.3 | 100 | 100 | 100 |
|  | 25 m | 46.7 | 51.7 | 60.0 | 60.0 | 65.0 | 66.7 | 65.0 | 93.3 | 93.3 | 95.0 | 98.3 | 98.3 | 100 | 100 |
|  | 50 m | 41.7 | 48.3 | 51.7 | 56.7 | 58.3 | 61.7 | 75.0 | 80.0 | 85.0 | 85 | 86.7 | 88.3 | 93.3 | 96.7 |
|  | 75 m | 30.0 | 31.7 | 41.7 | 38.3 | 40.0 | 46.7 | 55.0 | 63.3 | 75.0 | 76.7 | 80.0 | 81.7 | 88.3 | 95.0 |
|  | 100 m | 28.3 | 25.0 | 28.3 | 28.3 | 25.0 | 26.7 | 30.0 | 38.3 | 40.0 | 38.3 | 48.3 | 65.0 | 75.0 | 78.3 |
|  | **Total** | **46.0** | **48.0** | **53.0** | **54.3** | **55.0** | **58.7** | **63.3** | **74.3** | **78.0** | **78.3** | **82.3** | **86.7** | **91.3** | **94.0** |
|  |  |  |  |  |  |  |  |  |  |  |  |  |  |  |  |
| **Untreated control** | 10 m | 0 | 0 | 0 | 0 | 0 | 0 | 0 | 0 | 0 | 0 | 0 | 0 | 0 | 0 |
|  | 25 m | 0 | 0 | 0 | 0 | 0 | 0 | 0 | 0 | 0 | 0 | 0 | 0 | 0 | 0 |
|  | 50 m | 0 | 0 | 0 | 0 | 0 | 0 | 0 | 0 | 0 | 0 | 0 | 0 | 0 | 0 |
|  | 75 m | 0 | 0 | 0 | 0 | 0 | 0 | 0 | 0 | 0 | 0 | 0 | 0 | 0 | 0 |
|  | 100 m | 0 | 0 | 0 | 0 | 0 | 0 | 0 | 0 | 0 | 0 | 0 | 0 | 0 | 0 |
|  | **Total** | **0.0** | **0.0** | **0.0** | **0.0** | **0.0** | **0.0** | **0.0** | **0.0** | **0.0** | **0.0** | **0.0** | **0.0** | **0.0** | **0.0** |
| %, percentage; m, meter; min, minute; ULV, ultra-low volume. A total number of 300 adult females of each mosquito species were tested. | | | | | | | | | | | | | | | |
