# Supplementary material for: Semi-field evaluation of the space spray efficacy of Fludora Co-Max EW against wild insecticide-resistant Aedes aegypti and Culex quinquefasciatus mosquito populations from Abidjan, Côte d’Ivoire
Source: Parasit Vectors. 2023 Feb 2;16:47. doi: 10.1186/s13071-022-05572-5 (PMC9893543; doi:10.1186/s13071-022-05572-5)
Supplement: Supplementary file 10 — Additional file 10: Table S5. Mortality of the wild insecticide-resistant Aedes aegypti and Culex quinquefasciatus Abidjan strain mosquitoes exposed to Fludora Co-Max EW and K-Othrine EC using outdoor ULV space spray. ULV, ultra-low volume. [file 13071_2022_5572_MOESM10_ESM.docx]

| **Additional file 10: Table S5** Mortality of the wild insecticide-resistant *Aedes aegypti* and *Culex quinquefasciatus* Abidjan strains exposed to Fludora Co-Max EW and K-Othrine EC using outdoor ULV space spray | | | | | | | | | | | | | |
| --- | --- | --- | --- | --- | --- | --- | --- | --- | --- | --- | --- | --- | --- |
| **Mosquito species** | **Checkpoint** | **Fludora Co-Max EW** | | | | **K-Othrine EC** | | | | **Untreated control** | | | |
|  |  | **Dead** | **Alive** | **Mean (%)** | **SE** | **Dead** | **Alive** | **Mean (%)** | **SE** | **Dead** | **Alive** | **Mean (%)** | **SE** |
| *Aedes aegypti* | 10 m | 58 | 0 | 100.0 | 0.0 | 59 | 2 | 96.7 | 3.3 | 0 | 61 | 0.0 | 0.0 |
|  | 25 m | 59 | 1 | 98.3 | 1.7 | 45 | 15 | 75.0 | 5.8 | 1 | 59 | 1.7 | 1.7 |
|  | 50 m | 56 | 3 | 95.0 | 2.9 | 37 | 23 | 61.7 | 4.4 | 0 | 60 | 0.0 | 0.0 |
|  | 75 m | 51 | 9 | 85.0 | 2.9 | 38 | 22 | 63.3 | 4.4 | 0 | 58 | 0.0 | 0.0 |
|  | 100 m | 50 | 10 | 83.3 | 4.4 | 22 | 38 | 36.7 | 4.4 | 0 | 59 | 0.0 | 0.0 |
|  | **Total** | **274** | **23** | **92.3** | **2.1** | **201** | **100** | **66.7** | **5.5** | **1** | **297** | **0.3** | **0.3** |
|  |  |  |  |  |  |  |  |  |  |  |  |  |  |
| *Culex quinquefasciatus* | 10 m | 62 | 0 | 100.0 | 0.0 | 61 | 0 | 100.0 | 0.0 | 0 | 58 | 0.0 | 0.0 |
|  | 25 m | 63 | 0 | 100.0 | 0.0 | 59 | 0 | 100.0 | 0.0 | 0 | 62 | 0.0 | 0.0 |
|  | 50 m | 60 | 0 | 100.0 | 0.0 | 58 | 2 | 96.7 | 1.7 | 1 | 61 | 1.7 | 1.7 |
|  | 75 m | 60 | 0 | 100.0 | 0.0 | 53 | 4 | 93.3 | 4.4 | 0 | 57 | 0.0 | 0.0 |
|  | 100 m | 58 | 1 | 98.3 | 1.7 | 52 | 8 | 86.7 | 1.7 | 0 | 60 | 0.0 | 0.0 |
|  | **Total** | **303** | **1** | **99.7** | **0.3** | **283** | **14** | **95.3** | **1.6** | **1** | **298** | **0.3** | **0.3** |
| %: percentage, m: meter, SE: standard error, ULV: ultra-low volume | | | | | | | | | | | | | |
