# Supplementary material for: Semi-field evaluation of the space spray efficacy of Fludora Co-Max EW against wild insecticide-resistant Aedes aegypti and Culex quinquefasciatus mosquito populations from Abidjan, Côte d’Ivoire
Source: Parasit Vectors. 2023 Feb 2;16:47. doi: 10.1186/s13071-022-05572-5 (PMC9893543; doi:10.1186/s13071-022-05572-5)
Supplement: Supplementary file 11 — Additional file 11: Table S6. Knockdown rate at time intervals post-application in wild insecticide-resistant Aedes aegypti and Culex quinquefasciatus Abidjan strain mosquitoes exposed to outdoor TF space spray of Fludora Co-Max EW and K-Othrine EC. TF, thermal fogging. [file 13071_2022_5572_MOESM11_ESM.docx]

| **Additional file 11: Table S6** Knockdown rate (%) at time intervals post-application in wild insecticide-resistant *Aedes aegypti* and *Culex quinquefasciatus* Abidjan strains exposed to outdoor TF space spray of Fludora Co-Max EW and K-Othrine EC | | | | | | | | | | | | | | | |
| --- | --- | --- | --- | --- | --- | --- | --- | --- | --- | --- | --- | --- | --- | --- | --- |
| **Treatment arm** | **Checkpoint** | ***Aedes aegypti*** | | | | | | | ***Culex quinquefasciatus*** | | | | | | |
|  |  | **0 min** | **10 min** | **20 min** | **30 min** | **40 min** | **50 min** | **60 min** | **0 min** | **10 min** | **20 min** | **30 min** | **40 min** | **50 min** | **60 min** |
| **Fludora Co-Max EW** | 10 m | 95.0 | 96.7 | 100.0 | 100.0 | 100.0 | 100.0 | 100.0 | 98.3 | 98.3 | 98.3 | 100.0 | 100.0 | 100.0 | 100.0 |
|  | 25 m | 95.0 | 95.0 | 100.0 | 100.0 | 100.0 | 100.0 | 100.0 | 63.3 | 65.0 | 63.3 | 73.3 | 80.0 | 80.0 | 78.3 |
|  | 50 m | 91.7 | 88.3 | 90.0 | 86.7 | 95.0 | 95.0 | 100.0 | 33.3 | 31.7 | 33.3 | 48.3 | 53.3 | 63.3 | 63.3 |
|  | 75 m | 86.7 | 86.7 | 86.7 | 88.3 | 91.7 | 95.0 | 96.7 | 28.3 | 28.3 | 28.3 | 40.0 | 51.7 | 55.0 | 58.3 |
|  | 100 m | 71.7 | 71.7 | 78.3 | 78.3 | 75.0 | 83.3 | 88.3 | 28.3 | 28.3 | 28.3 | 40.0 | 46.7 | 50.0 | 51.7 |
|  | **Total** | **88.0** | **87.7** | **91.0** | **90.7** | **92.3** | **94.7** | **97.0** | **50.3** | **50.3** | **50.3** | **60.3** | **66.3** | **69.7** | **70.3** |
|  |  |  |  |  |  |  |  |  |  |  |  |  |  |  |  |
| **K-Othrine EC** | 10 m | 71.7 | 71.7 | 70.0 | 71.7 | 80.0 | 83.3 | 85.0 | 66.7 | 71.7 | 78.3 | 78.3 | 81.7 | 78.3 | 80.0 |
|  | 25 m | 66.7 | 70.0 | 55 | 63.3 | 66.7 | 63.3 | 61.7 | 51.7 | 51.7 | 45.0 | 56.7 | 68.3 | 80.0 | 83.3 |
|  | 50 m | 58.3 | 48.3 | 48.3 | 58.3 | 60.0 | 63.3 | 65.0 | 40.0 | 41.7 | 40.0 | 55.0 | 70.0 | 80.0 | 78.3 |
|  | 75 m | 60.0 | 53.3 | 51.7 | 60.0 | 58.3 | 65.0 | 68.3 | 43.3 | 41.7 | 45.0 | 61.7 | 68.3 | 76.7 | 81.7 |
|  | 100 m | 36.7 | 36.7 | 35.0 | 33.3 | 30.0 | 33.3 | 36.7 | 16.7 | 16.7 | 11.7 | 30.0 | 43.3 | 60.0 | 63.3 |
|  | **Total** | **58.7** | **56.0** | **52.0** | **57.3** | **59.0** | **61.6** | **63.3** | **43.7** | **44.7** | **44.0** | **56.3** | **66.3** | **75.0** | **77.3** |
|  |  |  |  |  |  |  |  |  |  |  |  |  |  |  |  |
| **Untreated control** | 10 m | 0 | 0 | 0 | 0 | 0 | 0 | 0 | 1.7 | 1.7 | 0 | 0 | 0 | 1.7 | 1.7 |
|  | 25 m | 0 | 0 | 0 | 0 | 0 | 0 | 0 | 0 | 0 | 0 | 0 | 0 | 0 | 0 |
|  | 50 m | 0 | 0 | 0 | 0 | 0 | 0 | 0 | 0 | 0 | 0 | 0 | 0 | 0 | 0 |
|  | 75 m | 0 | 0 | 0 | 0 | 0 | 0 | 0 | 0 | 0 | 0 | 1.7 | 0 | 1.7 | 1.7 |
|  | 100 m | 0 | 0 | 0 | 0 | 0 | 1.7 | 0 | 0 | 0 | 0 | 0 | 0 | 0 | 0 |
|  | **Total** | **0.0** | **0.0** | **0.0** | **0.0** | **0.0** | **0.3** | **0.0** | **0.3** | **0.3** | **0.0** | **0.3** | **0.0** | **0.7** | **0.7** |
| %, percentage; m, meter; min, minute; TF, thermal fogging. A total number of 300 adult females of each mosquito species were tested per treatment arm | | | | | | | | | | | | | | | |
