# Supplementary material for: Semi-field evaluation of the space spray efficacy of Fludora Co-Max EW against wild insecticide-resistant Aedes aegypti and Culex quinquefasciatus mosquito populations from Abidjan, Côte d’Ivoire
Source: Parasit Vectors. 2023 Feb 2;16:47. doi: 10.1186/s13071-022-05572-5 (PMC9893543; doi:10.1186/s13071-022-05572-5)
Supplement: Supplementary file 13 — Additional file 13: Table S8. Knockdown rate at time intervals post-application in wild insecticide-resistant Aedes aegypti and Culex quinquefasciatus Abidjan strain mosquitoes exposed to indoor ULV space spray of Fludora Co-Max EW and K-Othrine EC. [file 13071_2022_5572_MOESM13_ESM.docx]

| **Additional file 14: Table S9** Mortality of the wild insecticide-resistant *Aedes aegypti* and *Culex quinquefasciatus* Abidjan strains exposed to Fludora Co-Max EW and K-Othrine EC using indoor ULV space spray | | | | | | | | | | | | | |
| --- | --- | --- | --- | --- | --- | --- | --- | --- | --- | --- | --- | --- | --- |
| **Mosquito species** | **Checkpoint** | **Fludora Co-Max EW** | | | | **K-Othrine EC** | | | | **Untreated control** | | | |
|  |  | **Dead** | **Aive** | **Mean (%)** | **SE** | **Dead** | **Aive** | **Mean (%)** | **SE** | **Dead** | **Aive** | **Mean (%)** | **SE** |
| *Aedes aegypti* | Ceiling | 241 | 0 | 100 | 0 | 238 | 5 | 97.9 | 1.0 | 1 | 238 | 0.4 | 0.4 |
|  | Mid-height | 118 | 0 | 100 | 0 | 115 | 4 | 96.7 | 1.7 | 0 | 123 | 0.0 | 0.0 |
|  | Floor | 239 | 0 | 100 | 0 | 237 | 4 | 98.3 | 0.9 | 2 | 241 | 0.8 | 0.8 |
|  | **Total** | **598** | **0** | **100** | **0** | **590** | **13** | **97.8** | **0.6** | **3** | **602** | **0.5** | **0.4** |
|  |  |  |  |  |  |  |  |  |  |  |  |  |  |
| *Culex quinquefasciatus* | Ceiling | 246 | 0 | 100.0 | 0.0 | 242 | 0 | 100.0 | 0.0 | 3 | 236 | 1.3 | 0.9 |
|  | Mid-height | 121 | 0 | 100.0 | 0.0 | 124 | 0 | 100.0 | 0.0 | 0 | 116 | 0.0 | 0.0 |
|  | Floor | 244 | 0 | 100.0 | 0.0 | 238 | 0 | 100.0 | 0.0 | 0 | 239 | 0.0 | 0.0 |
|  | **Total** | **611** | **0** | **100.0** | **0.0** | **604** | **0** | **100.0** | **0.0** | **3** | **591** | **0.5** | **0.4** |
| %: percentage, SE: standard error, TF : thermal fogging | | | | | | | | | | | | | |
