# Supplementary material for: Semi-field evaluation of the space spray efficacy of Fludora Co-Max EW against wild insecticide-resistant Aedes aegypti and Culex quinquefasciatus mosquito populations from Abidjan, Côte d’Ivoire
Source: Parasit Vectors. 2023 Feb 2;16:47. doi: 10.1186/s13071-022-05572-5 (PMC9893543; doi:10.1186/s13071-022-05572-5)
Supplement: Supplementary file 14 — Additional file 14: Table S9. Mortality of the wild insecticide-resistant Aedes aegypti and Culex quinquefasciatus Abidjan strain mosquitoes exposed to Fludora Co-Max EW and K-Othrine EC using indoor ULV space spray. [file 13071_2022_5572_MOESM14_ESM.docx]

| **Additional file 15: Table 10** Knockdown rate (%) at time intervals post-application in wild insecticide-resistant *Aedes aegypti* and *Culex quinquefasciatus* Abidjan strains exposed to indoor TF space spray of Fludora Co-Max EW and K-Othrine EC | | | | | | | | | | | | | | | |
| --- | --- | --- | --- | --- | --- | --- | --- | --- | --- | --- | --- | --- | --- | --- | --- |
| **Treatment arm** | **Checkpoint** | ***Aedes aegypti*** | | | | | | | ***Culex quinquefasciatus*** | | | | | | |
|  |  | **0 min** | **10 min** | **20 min** | **30 min** | **40 min** | **50 min** | **60 min** | **0 min** | **10 min** | **20 min** | **30 min** | **40 min** | **50 min** | **60 min** |
| **Fludora Co-Max EW** | Ceiling | 100.0 | 100.0 | 100.0 | 100.0 | 100.0 | 100.0 | 100.0 | 100.0 | 100.0 | 100.0 | 100.0 | 100.0 | 100.0 | 100.0 |
|  | Mid-height | 100.0 | 100.0 | 100.0 | 100.0 | 100.0 | 100.0 | 100.0 | 99.2 | 99.2 | 99.2 | 100.0 | 100.0 | 100.0 | 100.0 |
|  | Floor | 100.0 | 100.0 | 100.0 | 100.0 | 100.0 | 100.0 | 100.0 | 100 | 100.0 | 100.0 | 100.0 | 100.0 | 100.0 | 100.0 |
|  | **Total** | **100.0** | **100.0** | **100.0** | **100.0** | **100.0** | **100.0** | **100.0** | **99.7** | **99.7** | **99.7** | **100.0** | **100.0** | **100.0** | **100.0** |
|  |  |  |  |  |  |  |  |  |  |  |  |  |  |  |  |
| **K-Othrine EC** | Ceiling | 90.8 | 93.8 | 97.5 | 97.9 | 98.3 | 99.2 | 98.8 | 81.7 | 83.8 | 86.3 | 91.7 | 92.1 | 93.3 | 93.8 |
|  | Mid-height | 89.2 | 90.8 | 96.7 | 98.3 | 98.3 | 99.2 | 100.0 | 78.3 | 86.7 | 88.3 | 95.0 | 95.0 | 95.0 | 95.8 |
|  | Floor | 92.1 | 91.7 | 94.2 | 96.3 | 97.1 | 98.8 | 98.8 | 95.8 | 92.9 | 95.8 | 97.9 | 99.2 | 100.0 | 100.0 |
|  | **Total** | **90.7** | **92.1** | **96.1** | **97.5** | **97.9** | **99.1** | **99.2** | **85.3** | **87.8** | **90.1** | **94.9** | **95.4** | **96.1** | **96.5** |
|  |  |  |  |  |  |  |  |  |  |  |  |  |  |  |  |
| **Untreated control** | Ceiling | 0 | 0 | 0 | 0 | 0 | 0 | 0 | 0 | 0 | 0 | 0 | 0 | 0 | 0 |
|  | Mid-height | 0 | 0 | 0 | 0 | 0 | 0 | 0 | 0 | 0 | 0 | 0 | 0 | 0 | 0 |
|  | Floor | 0 | 0 | 0 | 0 | 0 | 0 | 0 | 0 | 0 | 0 | 0 | 0 | 0 | 0 |
|  | **Total** | **0.0** | **0.0** | **0.0** | **0.0** | **0.0** | **0.0** | **0.0** | **0.0** | **0.0** | **0.0** | **0.0** | **0.0** | **0.0** | **0.0** |
| %, percentage; min, minute; TF, thermal fogging. A total number of 600 adult females of each mosquito species were tested per treatment arm | | | | | | | | | | | | | | | |
