# Supplementary material for: Semi-field evaluation of the space spray efficacy of Fludora Co-Max EW against wild insecticide-resistant Aedes aegypti and Culex quinquefasciatus mosquito populations from Abidjan, Côte d’Ivoire
Source: Parasit Vectors. 2023 Feb 2;16:47. doi: 10.1186/s13071-022-05572-5 (PMC9893543; doi:10.1186/s13071-022-05572-5)
Supplement: Supplementary file 15 — Additional file 15: Table S10. Knockdown rate at time intervals post-application in wild insecticide-resistant Aedes aegypti and Culex quinquefasciatus Abidjan strain mosquitoes exposed to indoor TF space spray of Fludora Co-Max EW and K-Othrine EC. [file 13071_2022_5572_MOESM15_ESM.docx]

| **Additional file 16: Table S11** Mortality of the wild insecticide-resistant *Aedes aegypti* and *Culex quinquefasciatus* Abidjan strains exposed to Fludora Co-Max EW and K-Othrine EC using indoor TF space spray | | | | | | | | | | | | | |
| --- | --- | --- | --- | --- | --- | --- | --- | --- | --- | --- | --- | --- | --- |
| **Mosquito species** | **Checkpoint** | **Fludora Co-Max EW** | | | | **K-Othrine EC** | | | | **Untreated control** | | | |
|  |  | **Dead** | **Aive** | **Mean (%)** | **SE** | **Dead** | **Aive** | **Mean (%)** | **SE** | **Dead** | **Aive** | **Mean (%)** | **SE** |
| *Aedes aegypti* | Ceiling | 241 | 0 | 100 | 0 | 238 | 2 | 99.2 | 0.6 | 0 | 242 | 0 | 0 |
|  | Mid-height | 120 | 0 | 100 | 0 | 118 | 0 | 100.0 | 0.0 | 1 | 119 | 0.8 | 0.8 |
|  | Floor | 242 | 0 | 100 | 0 | 238 | 2 | 99.2 | 0.6 | 0 | 240 | 0.0 | 0.0 |
|  | **Total** | **603** | **0** | **100** | **0** | **594** | **4** | **99.3** | **0.3** | **1** | **601** | **0.2** | **0.2** |
|  |  |  |  |  |  |  |  |  |  |  |  |  |  |
| *Culex quinquefasciatus* | Ceiling | 242 | 0 | 100.0 | 0.0 | 238 | 0 | 100.0 | 0.0 | 0 | 246 | 0.0 | 0.0 |
|  | Mid-height | 117 | 0 | 100.0 | 0.0 | 122 | 0 | 100.0 | 0.0 | 0 | 118 | 0.0 | 0.0 |
|  | Floor | 246 | 0 | 100.0 | 0.0 | 243 | 0 | 100.0 | 0.0 | 0 | 231 | 0.0 | 0.0 |
|  | **Total** | **605** | **0** | **100.0** | **0.0** | **603** | **0** | **100.0** | **0.0** | **0** | **595** | **0.0** | **0.0** |
| %: percentage, SE: standard error, TF : thermal fogging | | | | | | | | | | | | | |
