# Supplementary material for: Semi-field evaluation of the space spray efficacy of Fludora Co-Max EW against wild insecticide-resistant Aedes aegypti and Culex quinquefasciatus mosquito populations from Abidjan, Côte d’Ivoire
Source: Parasit Vectors. 2023 Feb 2;16:47. doi: 10.1186/s13071-022-05572-5 (PMC9893543; doi:10.1186/s13071-022-05572-5)
Supplement: Supplementary file 16 — Additional file 16: Table S11. Mortality of the wild insecticide-resistant Aedes aegypti and Culex quinquefasciatus Abidjan strain mosquitoes exposed to Fludora Co-Max EW and K-Othrine EC using indoor TF space spray. [file 13071_2022_5572_MOESM16_ESM.zip › 1-Additional file 12_Table S7_R1_29.10.2022.docx]

| **Additional file 12: Table S7** Mortality of the wild insecticide-resistant *Aedes aegypti* and *Culex quinquefasciatus* Abidjan strains exposed to Fludora Co-Max EW and K-Othrine EC using outdoor TF space spray | | | | | | | | | | | | | |
| --- | --- | --- | --- | --- | --- | --- | --- | --- | --- | --- | --- | --- | --- |
| **Mosquito species** | **Checkpoint** | **Fludora Co-Max EW** | | | | **K-Othrine EC** | | | | **Untreated control** | | | |
|  |  | **Dead** | **Alive** | **Mean (%)** | **SE** | **Dead** | **Alive** | **Mean (%)** | **SE** | **Dead** | **Alive** | **Mean (%)** | **SE** |
| *Aedes aegypti* | 10 m | 63 | 0 | 100.0 | 0.0 | 51 | 9 | 85.0 | 5.8 | 0 | 64 | 0.0 | 0.0 |
|  | 25 m | 55 | 5 | 91.7 | 7.6 | 44 | 16 | 73.3 | 1.7 | 0 | 58 | 0.0 | 0.0 |
|  | 50 m | 42 | 18 | 70.0 | 2.9 | 25 | 35 | 41.7 | 4.4 | 0 | 62 | 0.0 | 0.0 |
|  | 75 m | 36 | 24 | 60.0 | 2.9 | 28 | 32 | 46.7 | 4.4 | 0 | 59 | 0.0 | 0.0 |
|  | 100 m | 27 | 33 | 45.0 | 2.9 | 23 | 37 | 38.3 | 3.3 | 0 | 61 | 0.0 | 0.0 |
|  | **Total** | **223** | **80** | **73.3** | **5.5** | **171** | **129** | **57.0** | **5.2** | **0** | **304** | **0.0** | **0.0** |
|  |  |  |  |  |  |  |  |  |  |  |  |  |  |
| *Culex quinquefasciatus* | 10 m | 58 | 0 | 100.0 | 0.0 | 52 | 8 | 86.7 | 10.9 | 1 | 63 | 1.7 | 1.7 |
|  | 25 m | 59 | 0 | 100.0 | 0.0 | 58 | 3 | 95.0 | 5.0 | 0 | 60 | 0.0 | 0.0 |
|  | 50 m | 57 | 0 | 100.0 | 0.0 | 56 | 6 | 90.0 | 7.6 | 0 | 63 | 0.0 | 0.0 |
|  | 75 m | 59 | 2 | 96.7 | 3.3 | 52 | 8 | 86.7 | 1.7 | 1 | 61 | 1.7 | 1.7 |
|  | 100 m | 48 | 9 | 85.0 | 7.6 | 44 | 16 | 73.3 | 3.3 | 0 | 57 | 0.0 | 0.0 |
|  | **Total** | **281** | **11** | **96.3** | **2.1** | **262** | **41** | **86.3** | **3.1** | **2** | **304** | **0.7** | **0.5** |
| %: percentage, m: meter, SE: standard error, TF: thermal fogging | | | | | | | | | | | | | |
